# Adipocyte OGT governs diet-induced hyperphagia and obesity

**DOI:** 10.1038/s41467-018-07461-x

**Published:** 2018-11-30

**Authors:** Min-Dian Li, Nicholas B. Vera, Yunfan Yang, Bichen Zhang, Weiming Ni, Enida Ziso-Qejvanaj, Sheng Ding, Kaisi Zhang, Ruonan Yin, Simeng Wang, Xu Zhou, Ethan X. Fang, Tian Xu, Derek M. Erion, Xiaoyong Yang

**Affiliations:** 1 Program in Integrative Cell Signaling and Neurobiology of Metabolism and Department of Comparative Medicine, Yale University School of Medicine, New Haven, CT 06520 USA; 2 Department of Cellular and Molecular Physiology, Yale University School of Medicine, New Haven, CT 06520 USA; 3 Cardiovascular, Metabolic & Endocrine Disease Research Unit, Pfizer Worldwide Research & Development, Cambridge, MA 02139 USA; 4 Department of Genetics and Howard Hughes Medical Institute, Yale University School of Medicine, New Haven, CT 06520 USA; 5 Department of Immunobiology, Yale University School of Medicine, New Haven, CT USA; 6 Department of Statistics, Pennsylvania State University, University Park, PA 16802 USA; 7 Present Address: Department of Genetics and Complex Diseases and Sabri Ülker Center, Harvard T.H. Chan School of Public Health, 677 Huntington Avenue, Boston, MA 02115 USA

## Abstract

Palatable foods (fat and sweet) induce hyperphagia, and facilitate the development of obesity. Whether and how overnutrition increases appetite through the adipose-to-brain axis is unclear. *O*-linked beta-D-*N*-acetylglucosamine (O-GlcNAc) transferase (OGT) couples nutrient cues to O-GlcNAcylation of intracellular proteins at serine/threonine residues. Chronic dysregulation of O-GlcNAc signaling contributes to metabolic diseases. Here we show that adipocyte OGT is essential for high fat diet-induced hyperphagia, but is dispensable for baseline food intake. Adipocyte OGT stimulates hyperphagia by transcriptional activation of de novo lipid desaturation and accumulation of N-arachidonyl ethanolamine (AEA), an endogenous appetite-inducing cannabinoid (CB). Pharmacological manipulation of peripheral CB1 signaling regulates hyperphagia in an adipocyte OGT-dependent manner. These findings define adipocyte OGT as a fat sensor that regulates peripheral lipid signals, and uncover an unexpected adipose-to-brain axis to induce hyperphagia and obesity.

## Abstract (web summary)

Endocannabinoid signaling regulates food intake and is a potential therapeutic target for obesity. Here the authors show that adipocyte O-GlcNAc transferase (OGT) is required for high fat diet-induced hyperphagia via transcriptional activation of de novo lipid desaturation and accumulation of an endogenous appetite-inducing cannabinoid.

## Introduction

Overnutrition posed by palatable foods is a major culprit of the obesity epidemic worldwide. Numerous biological adaptation mechanisms exist to defend weight loss, which renders obesity notoriously difficult to treat through lifestyle changes^1,2^. Current anti-obesity drugs aim to reduce food intake and food cravings through targeting the general neurotransimitters and receptors. However, many of these drugs bring about intolerable risks of mental health problems and cardiovascular diseases^3^. Understanding what biological mechanisms define the set-point of body weight in an overnutritious environment is of paramount importance to develop new precision medicine that targets hyperphagia and obesity. Nutritional regulation of appetite is a critical mediator of overnutrition-induced obesity. Numerous studies have demonstrated the important role of brain nutrient sensors in the control of homeostatic appetite^4–6^. It is unclear whether and how adipose tissue senses overnutrition to induce hyperphagia^7^. Elucidating this adipose-to-brain axis will provide a valuable therapeutic window to treat obesity without the risk of psychiatric side effects.

Endocannabinoids are the endogenous ligands for the CB1 cannabinoid receptor. These lipid hormones are thought to link peripheral nutrient sensing to hyperphagia^8,9^. N-arachidonyl ethanolamine (AEA) is the prototypical endocannabinoid. The biosynthesis and degradation of AEA are catalyzed by N-acyl phosphatidylethanolamine phospholipase D (NAPE-PLD) and fatty acid amide hydrolase (FAAH), respectively, in various tissues, including brain, gut, and adipose tissue. Genetic and pharmacological studies on the CB1 receptor and FAAH show that the endocannabinoid system is essential for the regulation of food intake and body weight^8^. Remarkably, a periphery-restricted CB1 receptor antagonist is efficient in limiting food intake and body weight in rodents^10,11^. Decreased expression of FAAH and the CB1 receptor in adipose tissue is associated with human obesity^12^. Furthermore, mono-unsaturated lipid signals controlled by stearoyl-CoA desaturase (SCD) suppress FAAH and permit accumulation of AEA in peripheral tissues^13^. These studies suggest that the peripheral endocannabinoid system and lipid desaturation may contribute to diet-induced hyperphagia.

O-GlcNAc transferase (OGT) catalyzes the attachment of single *O-*linked beta-*N-*acetylglucosamine moieties to serine and threonine residues of intracellular proteins^14,15^. This dynamic and reversible protein O-GlcNAcylation enables diverse protein functions in response to cellular metabolic status. Hexosamine biosynthesis pathway (HBP) supplies the substrate UDP-GlcNAc for OGT. Chronic activation of the HBP and O-GlcNAc signaling has been implicated in the pathogenesis of cardiometabolic diseases^16,17^. Previous studies suggest that the HBP-OGT signaling affects leptin expression in adipose tissue in response to nutrient availability^18,19^. However, it is not known whether OGT in adipocytes is required for energy homeostasis, especially in an overnutritious environment.

Here we show that OGT in adipocytes mediates an adipose-to-brain signaling axis that enables hyperphagia and obesity during high-fat diet (HFD) feeding. Adipocyte OGT activates de novo lipid desaturation through transcriptional mechanisms, which facilitates the accumulation of endocannabinoids in adipose tissue. Peripheral tissue-derived endocannabinoids induces hyperphagia. These findings reveal that adipocyte OGT serves as a fat sensor that activates the peripheral endocannabinoid system, which in turn signals to the brain to induce hyperphagia and obesity.

## Results

### Adipocyte OGT drives diet-induced hyperphagia and obesity

**Fig. 1:**
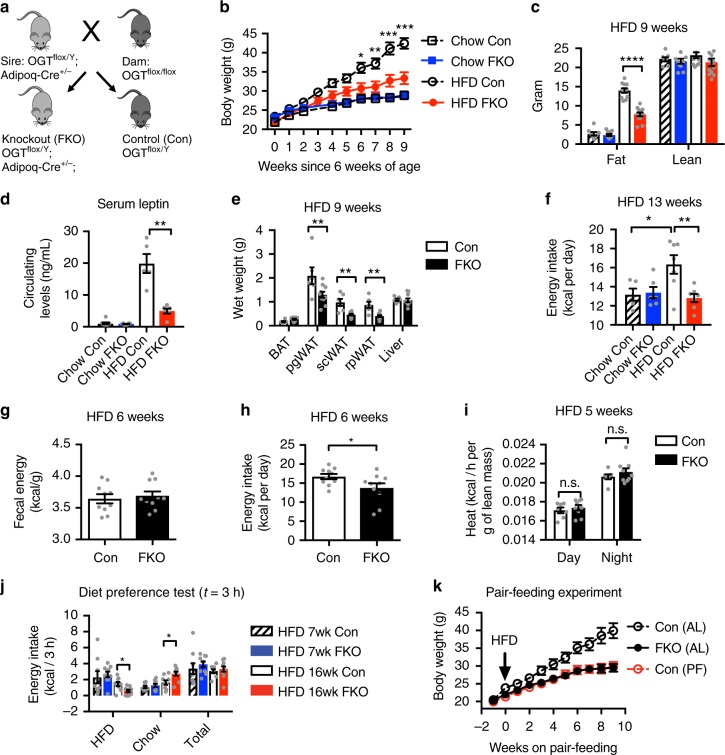
Adipocyte OGT drives diet-induced hyperphagia and obesity. **a** Genetic cross scheme. **b** Growth curves and **c** body composition of adipocyte-specific OGT knockout mice (FKO) and littermate control mice (Con) on chow (*n* = 8 (Con, dashed line with empty square/stripped bar) or 11 (FKO, blue line/bar)) or HFD (*n* = 11 (Con, dashed line with empty circle/empty bar) or 13 (FKO, red line/bar)). **d** Circulating levels of leptin and **e** wet tissue weights in male Con (*n* = 6) and FKO (*n* = 6) mice fed HFD for 9 weeks, or age-matched Con (*n* = 8) and FKO (*n* = 8) mice fed chow. BAT, interscapular brown adipose tissue. White adipose tissues include depots from perigonadal (pg), subcutaneous (sc), and retroperitoneal (rp) regions. **f** Energy intake of male mice on HFD (*n* = 7 per genotype) or Chow (*n* = 5 (Con) or *n* = 6 (FKO)) for 13 weeks. **g** Fecal energy content. **h** Daily energy intake. Male Con (*n* = 10) and FKO (*n* = 10) mice fed HFD for 6 weeks. **i** Energy expenditure of 5-week HFD-fed male Con (*n* = 7) and FKO (*n* = 9) mice. **j** Energy intake in a two-choice diet preference test. Male Con (*n* = 9) and FKO (*n* = 10) mice have been fed HFD for 7 or 16 weeks. **k** Growth curves of HFD-fed mice. Con (AL, ad libitum, *n* = 11), Con (PF, pair-fed to littermate FKO, *n* = 7), FKO (AL, *n* = 7). Data were presented as mean ± s.e.m. *P < 0.05, **P *<* 0.01, ***P *<* 0.001, ****P *<* 0.0001, post hoc Sidak’s tests (**b** (HFD Con vs. HFD FKO), **i**, **k** (Con PF vs. FKO AL)), Tukey’s tests (**c**, **d**, **f**), or two-tailed Student’s t-test (**e**, **g**, **h**, **j**)

To test the hypothesis that OGT in adipocytes regulates whole-body energy metabolism, we constructed adipocyte-specific OGT knockout (FKO) mice by crossing Adiponectin promoter-driven Cre mice with OGT^flox/flox^ mice (Fig. 1a). OGT FKO mice show dramatic reduction of OGT proteins and O-GlcNAc levels in adipose tissues, but exhibit no significant changes in OGT levels in the liver, muscle, and hypothalamus (Supplementary Figure 1). We fed OGT FKO and its littermate OGT^flox/flox^ control (Con) mice regular chow and HFD, respectively, and monitored the body weight and fat accumulation. The results show that OGT FKO mice gained remarkably less weight than Con mice on HFD but not on regular chow (week 9 on HFD body weight: Con 42.4 ± 1.38 g, FKO 33.4 ± 1.54 g, *P* *<* 0.0001; age- and sex-matched chow-fed Con 28.9 ± 0.76 g, FKO 28.6 ± 0.79 g, *P* *=* 0.999; One-way ANOVA *P* *<* 0.0001 followed by Tukey’s multiple comparisons test) (Fig. 1b). The decreased weight gain in HFD-fed FKO mice strongly associated with decreased accumulation of fat mass (Fig. 1c). In line with reduced fat mass, HFD-fed FKO mice had significantly reduced levels of serum leptin (Fig. 1d) and decreased wet weight of major white adipose depots (Fig. 1e). OGT deletion decreased leptin mRNA levels in adipose tissue on HFD, but not on chow diet (Supplementary Figure 2), which contributes to hypoleptinemia. Together, these results demonstrate that adipocyte OGT deletion abolishes HFD-induced obesity in mice.

The set-point of body weight is maintained by the balance between energy intake and energy expenditure^20^. To examine whether OGT deletion regulates any aspect of energy balance, we measured the expenditure and intake of energy in 13-week-HFD-fed or age-matched chow-fed OGT FKO mice. Remarkably, OGT deletion ablated HFD-induced hyperphagia, but had no significant impact on energy intake in mice raised on regular chow (Fig. 1f). OGT deletion did not alter the rectal temperature, energy expenditure, respiratory quotient, or physical activity after 12 weeks of HFD feeding (Supplementary Figure 3). Since body weight is a confounding factor for food intake measurement, we examined the energy balance in a turning time-point that OGT deletion started to abolish weight gain. At 6 weeks of HFD feeding, OGT deletion had no significant effect on the function of nutrient absorption (Fig. 1g), but significantly decreased energy intake (Fig. 1h). OGT deletion did not alter energy expenditure (Fig. 1i). HFD stimulates appetite in part through high palatability, as compared to low-fat diet^21^. In a two-choice test, we further observed that OGT FKO mice developed a preference toward regular chow after prolonged HFD feeding (16 weeks) (Fig. 1j). On regular chow, OGT FKO and Con mice exhibited indistinguishable diet preference (Supplementary Figure 4). These results show that adipocyte O-GlcNAc signaling senses dietary fat to induce hyperphagia and to promote preference toward highly palatable calorie-dense foods.

To test whether adipocyte OGT deletion abolishes diet-induced obesity through ablation of hyperphagia, we performed a pair-feeding experiment. At the age of 6 weeks, Con mice were pair-fed (PF) to weight-matched littermate OGT FKO mice in the HFD regimen. Another cohort of Con mice was fed ad libitum (AL) as a positive control for diet-induced obesity. We found that the body weight of pair-fed Con mice was indistinguishable from that of OGT FKO mice (Fig. 1k). As expected, pair-feeding normalized fat mass between FKO and Con mice (Supplementary Figure 5). These results strongly indicate that adipocyte O-GlcNAc signaling drives diet-induced obesity mainly through the induction of hyperphagia.

### Adipocyte OGT deletion ameliorates insulin resistance

**Fig. 2:**
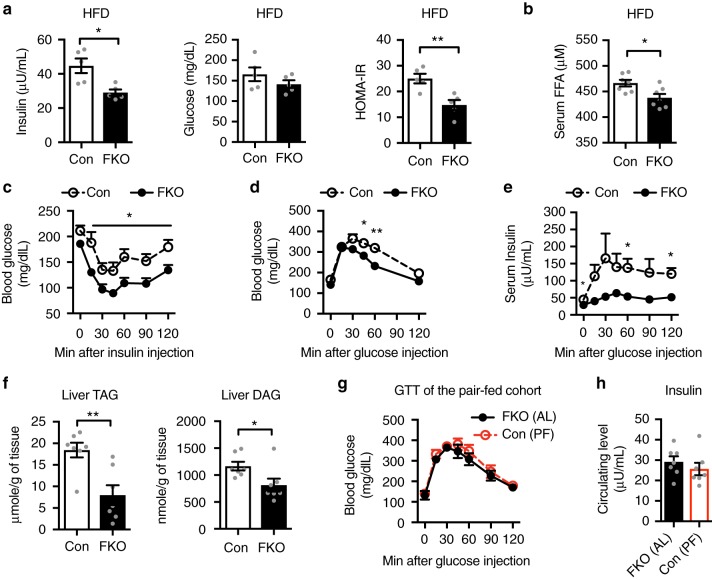
Adipocyte OGT drives diet-induced insulin resistance through obesity. **a** Basal insulin levels, glucose levels, HOMA-IR values in male Con (*n* = 5) and FKO (*n* = 5) mice fed HFD for 11 weeks. **b** Serum levels of free fatty acids (FFA). Mean ± s.e.m.: 466.4 ± 6.4 μM (Con, *n* = 7) vs. 437.8 ± 7.5 μM (FKO, *n* = 7). **c** Blood glucose response to intraperitoneal insulin tolerance test in male Con (*n* = 5) and FKO (*n* = 5) mice fed HFD for 12 weeks. **d**, ** e** Blood glucose (**d**) or blood insulin (**e**) response to intraperitoneal glucose tolerance test (GTT) in male Con (*n* = 5) and FKO (*n* = 5) mice fed HFD for 11 weeks. **f** Levels of triacylglycerol (TAG) and diacylglycerol (DAG) in livers of Con (*n* = 7) and FKO (*n* = 7) mice fed HFD for 22 weeks. **g**, **h** Blood glucose response to GTT (**g**), and basal insulin levels (**h**) in pair-fed (PF) Con (*n* = 7) and ad libitum (AL) FKO (*n* = 7) mice fed HFD for 9 weeks. Data were presented as mean ± s.e.m. *P < 0.05, **P *<* 0.01, two-tailed Student’s t-test (**a**, **b**, **f**, **h**), or post hoc Sidak’s tests (**c**–**e**, **g**)

To determine the metabolic effects of adipocyte OGT, we examined blood chemistry and glycemic control in OGT FKO mice fed HFD for 11–12 weeks. OGT deletion decreased hyperinsulinemia, an insulin resistance index (Fig. 2a), and hyperleptinemia (Supplementary Figure 6). OGT deletion did not alter total adiponectin levels in serum (Supplementary Figure 6). We profiled serum free fatty acids by targeted mass spectrometry (Supplementary Figure 7) and found that OGT deletion decreased circulating levels of free fatty acids (Fig. 2b). Furthermore, OGT FKO mice showed improved systemic insulin sensitivity (Fig. 2c), glucose tolerance (Fig. 2d), and insulin response to glucose load (Fig. 2e). Together, these data show that adipocyte OGT deletion ameliorates diet-induced insulin resistance.

Ectopic accumulation of diacylglycerols (DAG) results in obesity-associated insulin resistance^22^. Lipid profiling conducted in 22-week-HFD-fed Con and OGT FKO mice (Supplementary Figure 8) showed that OGT deletion decreased accumulation of triacylglycerol (TAG) and DAG in the liver (Fig. 2f). Furthermore, OGT FKO mice did not show any significant difference in glucose tolerance and circulating insulin levels when compared to body weight-matched pair-fed Con mice (Fig. 2g, h). Together, these results show that adipocyte OGT drives diet-induced insulin resistance through obesity.

### Transcriptomic and lipidomic changes in FKO adipose tissue

**Fig. 3:**
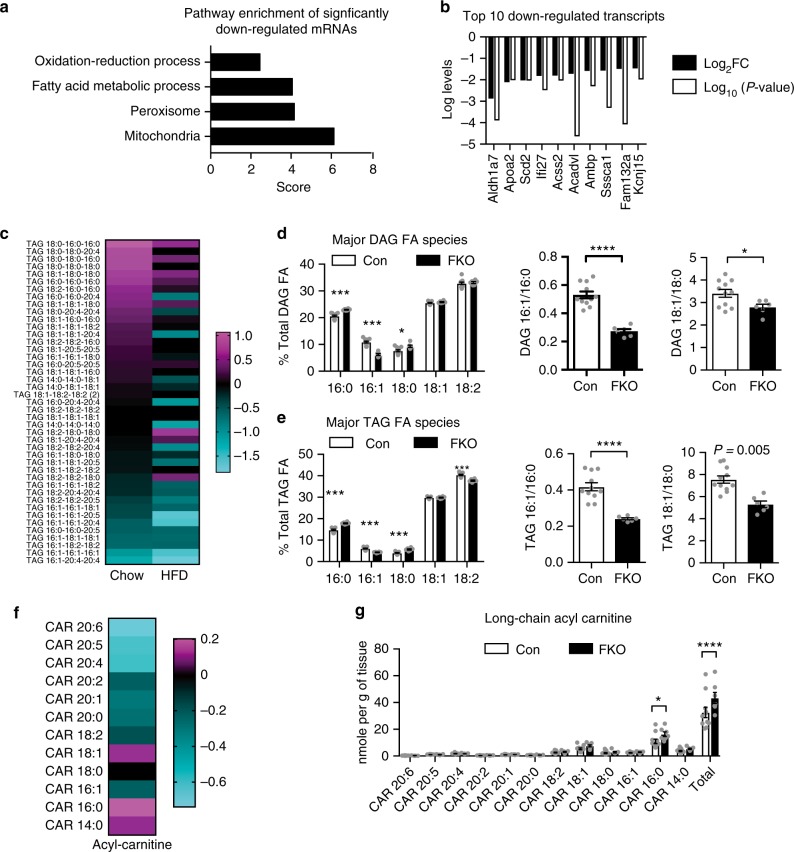
Transcriptomic and lipidomic analysis of adipose tissue from OGT FKO mice. **a** Pathway enrichment analysis of perigonadal white adipose tissue (pgWAT) from male Con (*n* = 5) and FKO (*n* = 3) mice fed HFD for 3 days. One hundred and thirty-four genes downregulated by OGT depletion were analyzed in the DAVID 7.8 platform. **b** Graph showing top 10 downregulated genes in adipose tissue. FC, fold-change of signals in FKO divided by signals in Con. **c** Heatmap of TAG lipid species in adipose tissue lipidome. Data were presented as log_2_ ratios of FKO/Con (Chow for 14–15 weeks, Con *n* = 11, FKO *n* = 6; age-matched HFD for 9 weeks, Con *n* = 7, FKO *n* = 8). **d**, **e** Content of major fatty acids in DAG and TAG in adipose tissue from chow-fed mice (*n* = 11 Con; *n* = 6 FKO), and lipid desaturation indexes (16:1/16:0, 18:1/18:0). **f**, **g** Heatmap (**f**) and Bar graphs (**g**) of the long-chain acyl-carnitine (CAR) profile of adipose tissue from mice described in panel (**d**, **e**). Data were presented as mean ± s.e.m. *P < 0.05, **P *<* 0.01, ***P < 0.001, ****P < 0.0001 two-tailed Student’s t-test (**d**, **e**), or post hoc Sidak’s multiple comparison test (**f**, **g**)

Histological analysis showed that OGT deletion did not alter adipocyte size in chow-fed OGT FKO mice, but significantly decreased the average size of adipocytes in mice on HFD for 9 weeks (Supplementary Figure 9). To determine the major impact of OGT depletion on adipocyte function, we profiled adipose tissue transcriptome from OGT FKO and Con mice challenged by HFD for 3 days. Pathway enrichment analysis of differentially regulated transcripts (including 157 downregulated and 149 upregulated genes) showed that transcriptional programs related to mitochondria, peroxisome, fatty acid metabolic process, and oxidation–reduction process were suppressed by OGT deletion (Fig. 3a), whereas mitochondria-related transcripts were upregulated (Supplementary Figure 10). Of the top 10 differentially regulated genes (Fig. 3b and Supplementary Figure 10), the presence of *Scd2* (stearoyl-CoA desaturase 2) suggests that de novo lipid desaturation may be impaired.

Lipidomic analysis of perigonadal white adipose tissue (pgWAT) from chow-fed and 9-week-HFD-fed Con and OGT FKO mice showed that levels of saturated fatty acids and mono-unsaturated fatty acids from TAG are differentially enriched (Fig. 3c). A similar pattern was observed in DAG species, but not in cholesterol esters or ceramides (Supplementary Figure 11). In fact, adipocyte OGT deletion increased palmitate (C16:0), stearate (C18:0) and decreased palmitoleate (C16:1) levels in both DAG (Fig. 3d) and TAG (Fig. 3e), which resulted in decreased lipid desaturation index (16:1/16:0 and 18:1/18:0, the latter is confounded by dietary C18:1). Acyl-carnitines are the substrates for fatty acid oxidation. Lipidomic profiling showed that OGT deletion increased the total level of long-chain acyl-carnitines, which is mainly attributed to the elevated level of palmitoyl-carnitine (Fig. 3f, g). This result shows that OGT FKO decreased fatty acid oxidation in adipocytes. Of note, adipocyte fatty acid oxidation is not required for weight gain during HFD feeding, though it is essential for adaptive thermogenesis^23,24^. Together, systematic analysis of the transcriptomic and lipidomic changes indicates that adipocyte OGT regulates lipid desaturation and oxidation.

### OGT trans-activates lipid desaturation in adipose tissue

**Fig. 4:**
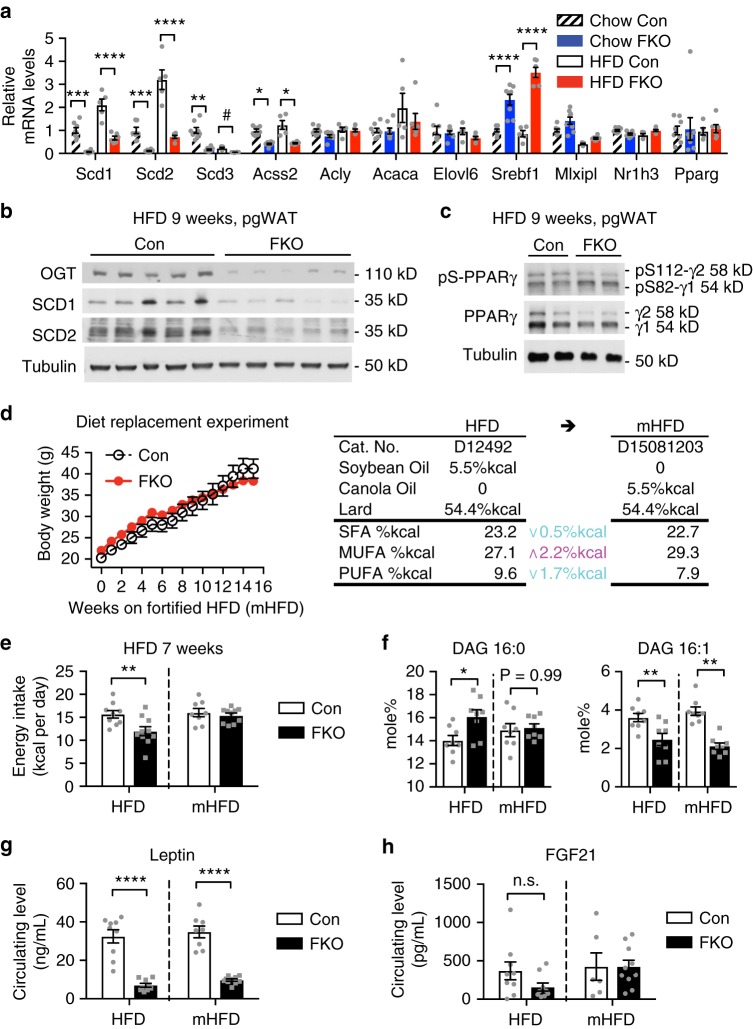
Adipocyte OGT drives diet-induced hyperphagia through transactivation of lipid desaturation in adipose tissue. **a** Gene expression analysis of de novo lipid desaturation and synthesis in adipose tissue (Chow for 15 weeks, Con *n* = 8, FKO *n* = 8; age-matched HFD for 9 weeks, Con n = 5, FKO *n* = 6). **b** Protein levels of SCD and OGT in adipose tissue. **c** Western blotting analysis in adipose tissue from 9-week-HFD-fed FKO and Con mice (Con *n* = 5, FKO *n* = 6, two representative biological replicates were shown). **d** Growth curves and **e** energy intake of (m)HFD-fed Con and FKO mice (*n* = 8–10 per group). mHFD denotes a mono-unsaturated fat-fortified high-fat diet that changes the saturated fat-rich soybean oil fraction into the mono-unsaturated fat-rich canola oil as illustrated in the diagram (**d**). **f** Contents of palmitate (C16:0) and palmitoleate (C16:1) DAG in adipose lipidome, and circulating levels of leptin (**g**), or of FGF21 (**h**) in mice fed HFD or mHFD for 15 weeks. Data were presented as mean ± s.e.m. n.s. not significant, *P < 0.05, **P *<* 0.01, ****P < 0.0001, post hoc Sidak’s tests (**a**, **e**–**h**), or ^#^*P* *<* 0.01 HFD Con vs. HFD FKO, two-tailed Student’s t-test (**a**). A dashed line in (**e**–**h**) indicates that data from HFD study and mHFD study are presented in a side-by-side manner, and inference may not be made between different diets

Next, we investigated whether adipose tissue lipid desaturation links OGT-mediated dietary fat sensing to hyperphagia. De novo desaturation of fatty acids is catalyzed by a family of stearoyl-CoA desaturases (*Scd1-4*, yet *Scd4* is not expressed in fat) in mice. We therefore tested whether adipocyte OGT promotes the expression of *Scd* genes. In the real-time quantitative PCR analysis of adipose tissue, we also included the catalytic and regulatory genes involved in de novo lipogenesis, as they are usually regulated together with *Scd* genes. The results show that OGT deletion decreased the expression of all *Scd* genes, *Acss2* (Acyl-coenzyme A synthetase short-chain family member 2, listed in Fig. 3b), but not that of *Acly* (ATP citrate lyase), *Acaca* (acetyl CoA carboxylase 1), or *Elovl6* (ELOVL family member 6, elongation of long-chain fatty acids) in the adipose tissue of HFD-fed mice or age-matched chow-fed mice (Fig. 4a). OGT deletion did not alter the expression of any lipogenic transcription factors we tested but *Srebf1* (Fig. 4a). Western blot analysis shows that OGT deletion decreased the protein levels of SCD1 and SCD2 in white adipose tissue (Fig. 4b and Supplementary Figure 12). A survey of expression of de novo lipogenic genes in both white and brown adipose tissues shows that OGT deletion selectively abolished the expression of *Scd* genes in white adipose tissues (Supplementary Figure 13). Consistent with this transcriptional profile, we observed that OGT deletion did not alter the protein levels of other enzymes in lipogenesis including fatty acid synthase (FASN) and ACACA (Supplementary Figure 14). Thus, adipocyte OGT is essential for expression of *Scd* genes in white adipose tissue.

### O-GlcNAcylation regulates adipose PPARγ

A substantial body of evidence demonstrates an essential role of PPARγ in transcriptional activation of *Scd* genes in mammals including humans^25,26^. It is known that PPARγ promotes lipid desaturation and the expression of *SCD* genes in cultured cells related to mammary glands^27^. We sought to verify that PPARγ is required for expression of *SCD* genes in adipose tissue in a mouse population and a genetic mouse model. Computational analysis of the hybrid mouse diversity panel strains showed that transcripts of *Scd1-3* and *Pparg* were positively correlated in the adipose tissue (Supplementary Figure 15). Indeed, adipose tissue-specific ablation of PPARγ decreased the expression of *Scd1* and *Scd2* in adipose tissue (Supplementary Figure 16). To determine whether OGT regulates PPARγ in vivo, we performed western blot analysis in adipose tissue of HFD-fed Con and FKO mice, and found that OGT deletion decreased the protein levels of PPARγ2 and increased the inhibitory Ser82 (or Ser112) phosphorylation of both PPARγ isoforms (Fig. 4c and Supplementary Figure 17). The isoform-specific regulation on PPARγ by OGT is important because *Pparg2* is restrictedly expressed in adipocytes and *Pparg1* is broadly expressed in tissues^28^. Together, these results show that OGT is required for PPARγ expression and activity in adipose tissue.

To provide mechanistic insights into OGT-mediated regulation of PPARγ, we performed site-directed mutagenesis studies on PPARγ2. Threonine (T) 54 is the only reported O-GlcNAc site on PPARγ1^29^, which corresponds to T84 of PPARγ2. We introduced the non-O-GlcNAcylable mutation (T-to-A) on T84 of PPARγ2, and determined the effects of T84A mutation on PPARγ2 O-GlcNAcylation and protein levels. 293A cells were transfected with expression vectors of PPARγ2 or T84A mutant, together with or without the expression vector of OGT, and subjected to immunoprecipitation. Western blot analysis of the immunoprecipitated proteins showed that PPARγ2 was O-GlcNAcylated by forced expression of OGT (Supplementary Figure 18). The T84A mutation dramatically decreased the expression and O-GlcNAcylation of PPARγ2, indicating that T84 O-GlcNAcylation is essential for PPARγ2 protein expression (Supplementary Figure 18). To determine whether T84 O-GlcNAcylation is required for PPARγ2 activity, we co-transfected 293A cells with expression vectors for PPARγ2 (or T84A mutant), RXR, and a PPRE-luciferase reporter, and examined the transcriptional activity of PPARγ2 in the baseline (vehicle-treated) and ligand-induced (Pioglitazone-treated) conditions. We found that T84A mutation abolished 74.4% of the baseline PPPAγ2 activity, and 44.5% of the ligand-induced activity (Supplementary Figure 19). Together, these results demonstrate an essential role of OGT in the regulation of PPARγ2 function via O-GlcNAcylation.

### OGT targets adipose lipid desaturation to enable hyperphagia

De novo lipid desaturation in adipose tissue is emerging as an important regulator of systematic metabolism^30,31^. To test whether de novo desaturation in adipose tissue links OGT to hyperphagia in HFD feeding, we fed FKO and Con mice with a modified HFD (mHFD), in which mono-unsaturated fat (predominantly made of oleate in fatty acid composition) is fortified while maintaining the total fat content (Fig. 4d). Con mice exhibited no preference between HFD and mHFD (Supplementary Figure 20). We observed that mHFD feeding rescued the obesity-associated phenotype of OGT FKO mice, including the body weight (Fig. 4d), fat mass (Supplementary Figure 21), energy intake (Fig. 4e), and contents of palmitate (C16:0) and oleate (C18:1) in DAG (Fig. 4f and Supplementary Figure 22). Since palmitoleate is low in diet, but mainly de novo desaturated from palmitate, mHFD did not normalize palmitoleate (C16:1) level in DAG (Fig. 4f). mHFD did not normalize the levels of PPARγ2 and SCD1 in adipose tissue (Supplementary Figure 23), supporting that mHFD acts downstream of PPARγ2. Interestingly, mHFD did not restore the decreased circulating level and tissue mRNA level of leptin in FKO mice (Fig. 4g and Supplementary Figure 24). This observation suggests that leptin is not a major mediator to OGT-controlled appetite regulation. Recently, fibroblast growth factor (FGF)-21 is arising as a periphery-to-brain hormone that reduces the diet preference for sweets and alcohol^32,33^. We found that circulating FGF21 levels were not significantly altered by OGT FKO in both HFD and mHFD feeding (Fig. 4h). Consistently, OGT FKO did not alter the transcript signature associated with PPARα activity in HFD-fed mice (Supplementary Figure 25). Our data suggests that the regulation of food intake and diet preference controlled by the adipose tissue OGT-SCD axis is not mediated by leptin or FGF21. Together, these results demonstrate that the OGT drives diet-induced hyperphagia and obesity through signals determined by adipose tissue lipid desaturation.

### OGT targets adipose endocannabinoids to enable hyperphagia

**Fig. 5:**
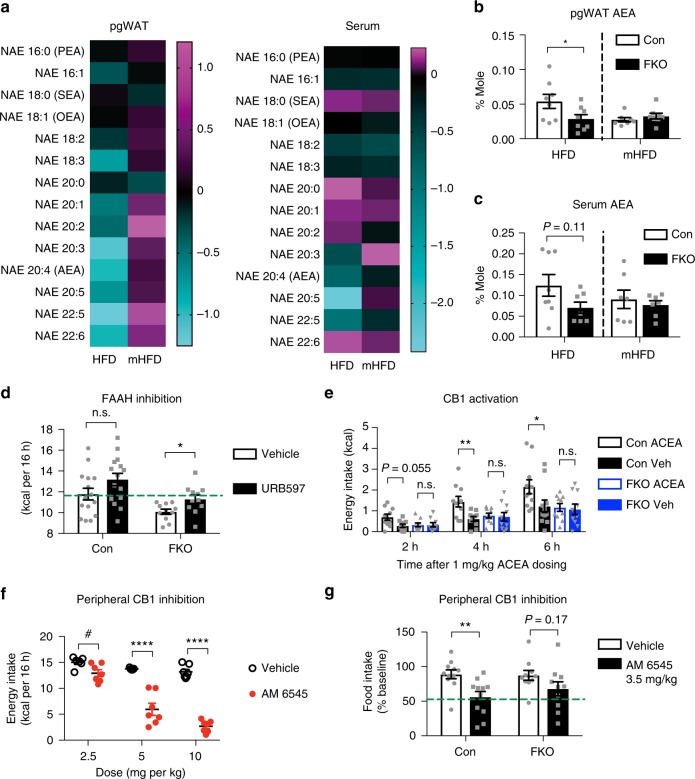
Adipose tissue endocannabinoid metabolism mediates the appetite-inducing effect of OGT. **a** Heatmap of *N*-acylethanolamine (NAE) profiles in adipose tissue (pgWAT) and serum from mice (*n* = 8 per group) fed HFD or mHFD for 14–15 weeks. Data has been processed to follow Gaussian distribution. Each box represents the log_2_ ratio of FKO/WT. **b**, **c** Levels of AEA in adipose tissue (**b**) and serum (**c**) from mice described above. **d** Energy intake of mice (*n* = 15 or 11) fed HFD for 10–13 weeks and dosed with FAAH inhibitor URB597 (0.5 mg/kg). n.s. not significant, *P *<* 0.05, multiple t-tests adjusted by the Holm–Sidak method. **e** Energy intake of mice (*n* = 11 or 10) fed HFD for 12–14 weeks and dosed with CB1 agonist ACEA (1 mg/kg). Mice were dosed at 8:30–9:00 a.m., and measured at indicated time points. n.s. not significant, *P < 0.05, **P *<* 0.01, repeat-measure post hoc Sidak’s tests. **f** Energy intake of mice (*n* = 7 per group) fed HFD for 13 weeks dosed with peripheral CB1 receptor blocker AM 6545 as indicated. Data were presented as mean ± s.e.m. n.s. not significant, *P < 0.05, **P *<* 0.01, ****P *<* 0.0001, post hoc Sidak’s tests, or ^#^*P* *<* 0.05, two-tailed Student’s t-test. **g** Food intake (% pre-injection baseline) of mice (*n* = 11 or 10) fed HFD for 10–11 weeks and dosed with CB1 antagonist AM 6545 (3.5 mg/kg). **P *<* 0.01, repeat-measure post hoc Sidak’s tests. A dashed line in (**b**, **c**) indicates that data from HFD study and mHFD study are presented in a side-by-side manner, and inference may not be made between different diets

Previous studies report that mono-unsaturated lipid signals generated by SCD suppress FAAH-controlled AEA degradation, and thus enable the activation of the endocannabinoid system in peripheral tissues^13^. To determine whether OGT-mediated lipid desaturation affects FAAH-controlled endocannabinoid degradation, we performed an unbiased lipid profiling of *N*-acylethanolamines (NAEs) in adipose tissue and serum from OGT FKO and Con mice. The results showed that OGT deletion decreased a plethora of NAE species derived from an arachidonyl backbone in HFD feeding, which was rescued by mHFD feeding (Fig. 5a). Particularly, OGT deletion significantly decreased AEA levels in adipose tissue from HFD-fed mice, but not in adipose tissue from mHFD-fed mice (Fig. 5b). OGT deletion may decrease circulating levels of AEA, but this effect is not statistically significant (Fig. 5c). The results of NAE profiling indicate that OGT suppresses FAAH-controlled endocannabinoid degradation in adipose tissue in a mono-unsaturated lipid-dependent manner.

Next, we determined the effects of OGT deletion on the expression of key components of endocannabinoid signaling in adipose tissue. OGT deletion did not alter the mRNA and proteins of *Faah*, and the mRNA levels of OEA receptors *Ppara* and *Gpr119*, and N-acyl taurine receptor *Trpv1* (Supplementary Figures 26, 27). Interestingly, OGT deletion induced the expression of *Nape-pld* and the CB1 receptor (*Cnr1*) in adipose tissue from HFD-fed mice (Supplementary Figure 26). These results support the notion that adipocyte OGT regulates the endocannabinoid system in adipose tissue.

To test whether increased FAAH activity in OGT FKO adipose tissue is required to abolish diet-induced hyperphagia, we intraperitoneally administered the FAAH inhibitor URB597 to HFD-fed mice and measured overnight energy intake. We found that inhibition of FAAH significantly increased the energy intake of OGT FKO mice, and restored the consumption of HFD to the level in vehicle-treated Con mice (Fig. 5d). In contrast, URB597 did not significantly induce energy intake in Con mice at the dose we tested (Fig. 5d).

To determine whether signaling processes downstream of FAAH-controlled AEA degradation contribute to the appetite-inducing effect of OGT, we investigated the effect of a CB1 agonist or antagonist on energy intake in OGT FKO mice. On one hand, administration of the CB1 agonist, arachidonyl-2′-chloroethylamide (ACEA), significantly increased the energy intake of Con mice, but did not affect that of OGT FKO mice (Fig. 5e). On the other hand, we took advantage of the CB1 antagonist AM 6545 to inhibit peripheral CB1 signaling. Remarkably, peripheral inhibition of CB1 signaling decreased the energy intake of HFD-fed mice in a dose-dependent manner (Fig. 5f), even at doses (2.5 mg per kg and 5 mg per kg) that are not effective during chow feeding^10^. As expected, peripheral inhibition of CB1 signaling decreased the energy intake of Con mice, but did not significantly affect that of OGT FKO mice (Fig. 5g). Clearly, OGT FKO mice are responsive to FAAH inhibition, and resistant to pharmacological manipulation of CB1 signaling, which strongly supports the conclusion that suppression of peripheral AEA degradation mediates adipocyte OGT-activated axis to develop hyperphagia.

## Discussion

**Fig. 6:**
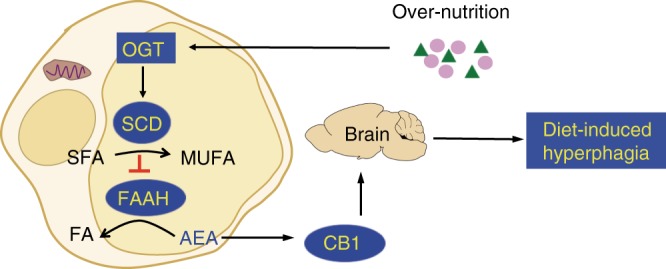
A working model for appetite regulation in obesity. The fat-sensing adipose tissue-to-brain axis induces hyperphagia when palatable foods are available. This appetite-inducing axis in adipose tissue is activated by the fat-sensing module OGT, which trans-activates expression of the lipid desaturation module SCD. SCD facilitates the accumulation of the appetite-inducing module AEA, which activates peripheral CB1 signaling to induce hyperphagia

In this study, our analysis of a mouse model with adipocyte-specific ablation of OGT uncovers an unexpected fat-sensing OGT-SCD axis and defines its pivotal role in linking peripheral AEA metabolism with hyperphagia and obesity (Fig. 6). Intracellular O-GlcNAc signaling has multiple target proteins. The unbiased transcriptomic and lipidomic studies prioritized our focus to SCD-controlled de novo lipid desaturation process. We have shown that OGT activates expression of all *Scd* isoforms expressed in adipose tissue (Fig. 4a and Supplementary Figure 13). OGT FKO thus emulates a genetic knockdown model for *Scd* genes in adipose tissue. Previously, adipocyte/macrophage-specific deletion of *Scd1* showed a trendy but not statistically significant reduction on diet-induced obesity^34^. Germline Scd1 deletion is hypermetabolic and adaptively hyperphagic, due to skin disintegration-associated heat loss^35–37^. The effect of *Scd1* deletion on hyperphagia may be masked by other isoforms and different sources, or the ectopic effect of the aP2-Cre line^38^. It is possible that mono-unsaturated lipid signals that regulate FAAH activity are more dependent on SCD2 (perhaps SCD3) than SCD1. The success of mHFD feeding studies in restoring hyperphagia, fat mass, and body weight in OGT FKO mice strongly supports the conclusion that adipocyte OGT trans-activates lipid desaturation to induce hyperphagia during nutrient surplus. A recent randomized trial shows that Mediterranean diet, which is rich in mono-unsaturated fat and dietary fiber, prevents cardiometabolic diseases with weak impact on obesity^39,40^. The seemingly discrepancy between animal models and human studies may originate from the difference in diet composition (e.g., dietary fiber), food quality, and genetics. Nevertheless, our studies indicate an essential role of endogenous lipid desaturation in the etiology of appetite regulation in obesity.

Marijuana and endocannabinoids are long known to induce appetite and weight gain^8^. Targeting the CB1 cannabinoid receptor by antagonists hold tremendous potential for controlling severe obesity, though the pleiotropic effects on cognitive functions of these drugs (e.g., anxiety and depression, or even suicide attempts) limit their clinic use^3^. In our work, a combination of molecular, genetic, and pharmacological evidence demonstrate that adipocyte OGT senses dietary fat and suppresses AEA degradation to activate hyperphagia. This adipose-to-brain axis may be complementary to or upstream of leptin signaling in the hypothalamus. However, we do not know the exact tissues and cell types that are engaged by adipose tissue AEA. It is generally agreed that the biological effects of AEA are spatially limited to cells nearby^41^. Adipose tissue AEA signaling promotes lipogenesis, and is connected to the nervous system^42,43^. It is highly possible that adipose tissue AEA may engage the CB1 receptor in adipose tissue to regulate feeding behavior. Indeed, it has been documented that obesity-related upregulation of the CB1 receptor in adipose tissue may sensitize the appetite-inducer effect of AEA in rodents^44^. A recent study using an inducible knockout model reports that adipocyte CB1 modulates energy expenditure and modulates body weight on both chow and HFD feeding^45^. While our study and this study use different knockout models, these findings suggest that adipocyte CB1 may be the principal mediator of appetite-inducing OGT-AEA axis. We did not observe any effect of OGT FKO on the anorexigenic endocannabinoid oleoylethanolamine (OEA) and its receptors such as *Ppara* and *Gpr119* in adipose tissue (Fig. 5a and Supplementary Figure 26). Together, our studies define peripheral CB1 signaling as an essential mediator in the etiology of hyperphagia, and indicate an avenue for anti-obesity drug development.

In the past decade, multiple laboratories including us have demonstrated the tremendous therapeutic potential of systematic inhibiting OGT in obesity-related cardiometabolic diseases. So far, all tissue-specific OGT knockout mouse models have shown consistent and clinically meaningful benefits in cardiometabolic diseases^15^. In liver, OGT inhibits insulin signaling^46^ and increases glucose production^47,48^. In heart, OGT mediates diabetic hyperglycemia-induced arrhythmia^49^. In hunger neurons, OGT inhibits browning of white fat and energy expenditure in obesity^50^. In paraventricular neurons, OGT promotes satiety during regular chow feeding^51^, yet it is not known this mechanism is engaged in HFD feeding. Here, we showed that adipocyte OGT is engaged during nutrient surplus, and is essential in the etiology of hyperphagia and obesity. Specifically, targeting adipocyte OGT and its controlled downstream signaling pathways may treat obesity in a precise manner that would not interfere with mental health and homeostatic feeding. Currently, OGT inhibitors are developing at a rapid speed^52–54^, and human OGT structure has been revealed recently^55^. Hence, a systematic approach to inhibit OGT represents a promising strategy to stop the epidemic of obesity and cardiometabolic diseases.

## Methods

### Animal studies

C57BL/6J.Adiponectin-Cre strain is bred to OGT^flox/flox^ stain to generate Adiponectin-Cre; OGT^flox/Y^ (OGT FKO or FKO) line and wildtype litermates (OGT^flox/Y^, Con). Male mice were maintained under 12 h/12 h light/dark cycle under constant conditions of temperature (22 °C) and humidity with free access to food (Teklad Global 18% protein rodent diet 2018S) and water. For HFD feeding experiments, 6–7 weeks old male mice were fed with HFD (60 kcal%, Research Diets Inc. D12492) or custom made isocaloric high-mono-unsaturated fatty acid (MUFA, 60 kcal%, Research Diets Inc. D15081203) diets for up to 24 weeks. Tissues were collected from euthanized 6-h-fasted mice. All procedures have been approved by the Institutional Animal Care and Use Committee of Yale University.

### Pair-feeding studies

For pair-feeding experiments, 6 weeks old male mice (*n* = 11) were housed individually. Pair-fed with HFD study was performed from 7 weeks of age for 12 weeks. Con mice were paired with age- and weight-matched OGT FKO mice. For the pair-fed Con group, animals were pair-fed to the amount of daily food intake consumed by the ad libitum groups of OGT FKO mice the previous day. The amount of daily food was given at 9:00–10:00 a.m. Food intake was measured daily for the period of the pair-fed experiment and body weight was measured every week.

### Metabolic phenotyping assays

Body weight was monitored weekly by a digital precision scale (accuracy 0.1 g). Body composition (fat and lean mass) was assayed by an EchoMRI system. *Energy intake analysis*: Mice were individually housed at least 1 week prior to the energy intake analysis, for environmental habituation. Energy intake was calculated as consumed food quantity timed by energy density (3.1 kcal/g for regular chow or 5.24 kcal/g for HFD). Food consumption was manually weighed by a digital precision scale (accuracy 0.01 g). Food pellets were layed on the bedding of cages to avoid smashing. Two-choice preference test was performed by weighing and assigning HFD and regular chow food pellets to the bedding of each cage that housed a single mouse at 6:45 p.m., and weighing the food weight at 10:00 p.m. (3 h after light-out) and 11:00 a.m. (16 h after light-out). Energy intake was produced as described above. Pharmacological interventions were performed as follows. Reagents (specifics can be found in the Method section 'Reagents') were administered intraperitoneally 30 min before foods were provided by following either an alternating dosing regimen where repeat-measure statistic analysis applies, or a regular dosing regimen as indicated. Food pellets were weighed at 0, 3, and 16 h after provided. For ACEA, food pellets were weighed at 0, 2, 4, and 6 h after provided. *Energy metabolism analysis*: Mice were acclimated in metabolic chambers (Promethion Systems, Sable Systems International) for 5 days and then gas exchange, and ambulatory activity (expressed as the number of beam breaks) were recorded continuously for another 2 days. Heat production was calculated and adjusted to lean body mass. Fecal energy content is measure by bomb calorimetry. In all, 400–500 mg of fecal samples were dried at 37 °C for 48 h and analyzed in IKA® calorimeter system (C5000 control). The calorimeter energy equivalent factor was determined by benzoic acid standards.

### Glucose tolerance test and insulin tolerance test

To determine glucose tolerance, 16-h-fasted mice were intraperitoneally administered with glucose (1 g/kg of body weight). To determine insulin sensitivity, 6-h-fasted mice were intraperitoneally administered with insulin (1 U/kg of body weight, Humulin R, Lilly). Blood glucose from tail-vein blood was quantified by a NovaMax glucometer at designated time after administration.

### Serum biochemical analysis

Serum levels of insulin, leptin, adiponectin, and FGF21 were assayed by ELISA kits (EMD Millipore *EZRMI-13K, EZML-82K*; R&D Systems *MRP300*, *MF2100*).

### Lipid profiling

All mouse adipose tissue samples were homogenized in methanol:water (1:1, v/v) using the Qiagen tissue lyser. Following homogenization, lipids were extracted from 5 μL homogenate with 2000 μL of the extraction solvent: dichloromethane:isopropanol:methanol (25:10:65, v/v/v). Lipid extracts were then analyzed by UPLC-MS/MS using a Waters Acquity UPLC coupled to a Sciex QTRAP 5500 mass spectrometer. Lipid classes were separated by reversed-phase chromatography on a Waters Acquity UPLC BEH300 C4 column, 1.7 μm, 2.1 × 50 mm. Lipid species were then analyzed on the mass spectrometer using positive ion electrospray ionization in the multiple reaction-monitoring (MRM) mode. LC chromatogram peak integration was performed with Sciex MultiQuant software. All data reduction was performed with in-house software in Pfizer Inc.

### Free fatty acid profiling

Serum free fatty acids were extracted with methanol:water (80:20 v/v) containing the following internal standard at 200 nM: heptadecanoic acid. Free fatty acid extracts were then analyzed by UPLC-MS using a Waters Acquity UPLC coupled to a Thermo LTQ Orbitrap Velos mass spectrometer. Free fatty acid species were separated by reversed-phase chromatography on a Waters Acquity UPLC BEH C18 column, 1.7 μm, 2.1 × 50 mm. Free fatty acids were then analyzed on the mass spectrometer using negative ion electrospray ionization in the full scan MS mode. Free fatty acid identification and LC chromatogram peak integration was performed with Thermo Sieve software. All data reduction was performed with in-house software in Pfizer Inc.

### *N*-acylethanolamine profiling: adipose tissue

All mouse adipose tissue tissue samples were homogenized in methanol:water (1:1, v/v) using the Qiagen tissue lyser. Following homogenization, NAEs were extracted from 100 μL homogenate with 2.0 mL of chloroform:methanol (2:1, v/v) containing 0.01% BHT in a glass vessel, followed by the addition of C17:0 NAE internal standard solution and 250 μL water. The glass vessel was vortexed for 30 s and centrifuged at 4000 × *g* (4 °C) for 20 min. The bottom organic phase was transferred to another glass vessel, dried down under nitrogen (N_2_), resuspended in 200 μL Chloroform:Methanol (2:1, v/v), and transferred to a glass LC vial for analysis. Serum NsAEs were extracted from 100 μL mouse plasma with 2.0 mL of Chloroform:Methanol (2:1, v/v) containing 0.01% BHT in a glass vessel, followed by the addition of C17:0 NAE internal standard solution and 250 μL water. The glass vessel was vortexed for 30 s and centrifuged at 4000 × *g* (4 °C) for 20 min. The bottom organic phase was transferred to another glass vessel, dried down under nitrogen (N_2_), resuspended in 200 μL chloroform:methanol (2:1, v/v), and transferred to a glass LC vial for analysis. NAE extracts were then analyzed by UPLC-MS/MS using a Waters Acquity UPLC coupled to a Sciex API4000 mass spectrometer. NAE species were separated by reversed-phase chromatography on an EMD Hibar Lichrosorb C8 column, 5 μm, 4 × 125 mm. NAE species were then analyzed on the mass spectrometer using positive ion electrospray ionization in the MRM mode. LC chromatogram peak integration was performed with Sciex MultiQuant software. All data reduction was performed with Microsoft Excel.

### Microarray analysis

Total RNA was purified from perigonadal white adipose tissue of WT (*n* = 3) and OGT FKO (*n* = 5) mice that had been challenged on HFD for 3 days. RNA was converted to complementary DNA and hybridized on Illumina MouseRef-8 v2.0 expression beadchip. Quantile normalized Illumina microarray data were used for gene expression analysis. Significant expression change was determined for each gene with both *P*-value < 0.01 and fold expression > 1.5-fold. The *P*-value for each comparison was determined with Matlab ttest2 function. Overall, we identified 149 genes and 157 genes with significantly changes in the perigonadal white adipose tissue.

### RNA extraction and real-time quantitative PCR

Tissue RNAs were isolated using TRIzol Reagent (Invitrogen). Complementary DNA (cDNA) was synthesized using the iScript cDNA Synthesis Kit (Bio-Rad). cDNA was amplified and analyzed using iQ SYBR Green Supermix (Bio-Rad) and the LightCycler 480 Instrument II (Roche) as described previously^56^. Q-PCR data were normalized to u36b4. Primer sequences were listed in Supplementary Table 1. Experiments were repeated twice.

### Western blot assay

α-O-GlcNAc (RL2, Abcam, ab2739, 1:1000; CTD110.6, Cell Signaling, #12938, 1:10,000), α-OGT (Cell Signaling, #5368, 1:1000), α-SCD1 (ThermoFisher, PA5-17409, 1:1000), α-SCD2 (G15, Santa Cruz, sc-14722, 1:200), α-PPARγ (D69, Cell Signaling, 2430, 1:1000), α-phospho-PPARγ (AW504, Millipore, 04-816, 1:1000), α-Acetyl CoA carboxylase 1 (Cell Signaling, 4190, 1:1000), α-Fatty acid synthase (Novus, NB400-114, 1:1000), α-Nape-pld (Abcam, ab95397, 1:1000), α-FAAH (Abcam, ab54615, 1:1000), α-FLAG (M2, Sigma, A8592, 1:10,000), α-GAPDH (FL-335, Santa Cruz, sc-25778, 1:1000), and α-alpha-Tubulin (B-5-1-2, Sigma, T5168, 1:1000) were purchased from vendors. Tissues were lysed in buffer containing 1% Nonidet P-40, 50 mM Tris-HCl, 0.1 mM EDTA, 150 mM NaCl, proteinase inhibitors and protein phosphatase inhibitors. Thirty micrograms of protein lysate were electrophoresed on SDS-PAGE gels and transferred to methanol-activated PVDF membranes. Primary antibodies were incubated at 4 °C over the night. Western blotting was visualized by peroxidase conjugated secondary antibodies and ECL chemiluminescent substrate. Uncropped western blots images were shown in Supplementary Fig. 12 and Suplementery Fig. 17. O-GlcNAc signals detected by CTD110.6 antibody were visualized by SuperSignal ECL (ThermoFisher, 34095). Experiments were repeated three times.

### Plasmids

PPRE X3-TK-luc was a gift from Bruce Spiegelman (Addgene plasmid # 1015). pcDNA flag PPAR gamma was a gift from Bruce Spiegelman (Addgene plasmid # 8895). pSV Sport RXR alpha was a gift from Bruce Spiegelman (Addgene plasmid # 8882). Other plasmids include pcDNA3 and pGL3/ CMV-Renilla luc (Promega E2261).

### Cell culture

293A cells (ThermoFisher cat# R70507) were maintained in high glucose Dulbecco’s modified Eagle’s medium (DMEM) with 10% fetal bovine serum (FBS). Cell line was authenticated by microscope-assisted morphology check, growth curve analysis, and mycoplasma detection.

### Transactivation assay

In a 96-well microplate, 293A cells were transfected with 10 ng pGL3/PPRE, 1 ng pRL CMV, 10 ng pSV Sport/RXRA, and 10 ng of pcDNA6 or pcDNA3/PPARγ2 (WT or site-directed mutants) in hexaplicates. Forty-eight hours later, cells were treated with 10 μM pioglitazone (Cayman #71745) or vehicle (DMSO) for 6 h. Cells were lysed in 50 μL of 1x Passive Lysis Buffer (Promega) for 15 min on ice. Ten microliters of lysates were aliquoted to a new white-bottom 96-well microplate, and assayed by the Dual-Luciferase Reporter Assay System (Promega) by following the manufacturer’s instructions with minor modification (reaction buffer volume down-sized to 50 μL per 10 μL of lysates). The integration time for luminescence signals is 140 ms. Promoter activity is presented as Firefly luminescence intensity normalized to Renilla luminescence intensity. Promoter activity from vehicle-treated control vector-transfected cells was set to 1. Experiments were repeated for four times.

### Reagents

URB597 (0.5 and 5 mg/kg, Cayman cat# 10046) was dissolved in DMSO:ethanol (1:1 v/v) at a concentration of 6.25 mg/mL. It was diluted to sterile vehicle solution (5% Tween-80, 5% PEG (average molecular weight 200) in PBS) at a concentration of 0.1 and 1.0 mg/mL, respectively. ACEA (1 mg/kg, TOCRIS cat# 1319) was supplied as a 5 mg/mL solution in methyl acetate. It was concentrated by a centrifugal concentrator (SpeedVac Plus SC110A), re-dissolved in DMSO and diluted to sterile vehicle solution (5% Tween-80 in saline) at a concentration of 0.2 mg/mL. AM 6545 (2.5, 3.5, 5, 10 mg/kg, Cayman cat# 16316) was dissolved in DMSO, first diluted to Tween-80 at a 1:2 ratio, and then to saline to achieve a concentration of 0.5, 0.7, 1, 2 mg/mL. Recombinant mouse leptin protein (5 mg/kg, R&D Systems cat# 498-OB) was reconstituted at 1 mg/mL in sterile 20 mM Tris-HCl, pH 8.0. The reagents were delivered via intraperitoneal injection to mice.

### Gene network analysis

To further study how the genes interact with each other, we adopt Gaussian graphical model^57^ to estimate the conditional independences among the genes expressed in gonadal white adipose tissues from the hybrid mouse diversity panel (GSE64768)^58^. The microarray dataset was originally collected for genome-wide association studies to identify genetic loci associated with obesity. The data is pre-processed by standardizing the mean and standard deviation of each probe to 0 and 1, respectively, and the expression levels of different probes are averaged if the probes correspond to a same gene. To ensure that the estimated graph achieves the desired sparse level, the tuning parameter in the Gaussian graphical model is chosen to be 0.3.

### Statistical analysis

All statistical tests were analyzed by two-tailed Student’s t-test for comparison of two groups, or analysis of variance (ANOVA) (with post hoc comparisons using Sidak’s test) using a statistical software package (GraphPad Prism 6.0) for comparison of multiple groups. Statistical tests regarding body weight and energy balance were assessed after confirming that the data met appropriate assumptions (normality, homogenous variance, and independent sampling). Normality was examined by the D’Agostino–Pearson omnibus test, and the Shapiro–Wilk test (GraphPad Prism 6.0). No sample was excluded from the reported analyses. Mice were excluded from the analyses when they died by accident during the alternating dosing regimens, so as to keep the dataset amenable to repeat-measure post hoc analyses. Exclusion criteria were pre-established, i.e., sick or dead animals after pharmacological intervention were excluded from data analysis. In genetic intervention studies, allocation was not random, but littermate control animals were used to control covariates, such as age and sex. In pharmacological intervention studies, allocation was randomized. Investigators were not blinded to group allocation in data collection/analysis based on general practice, except when performing pharmacological intervention studies. The sample size (the number of mice, or wells of cells) was determined by Power analysis (Alpha = 0.05, Power = 0.80). *P-*value < 0.05 was considered statistically significant. All data are mean ± s.e.m.

## Electronic supplementary material

Supplementary Information

## Data Availability

The datasets generated during and/or analyzed during the current study are available from the corresponding author on reasonable request. Microarray data that support the findings of this study have been deposited in the Gene Expression Omnibus (GEO) with the accession codes GSE111735.
