# Supplementary material for: Adipocyte OGT governs diet-induced hyperphagia and obesity
Source: Nat Commun. 2018 Nov 30;9:5103. doi: 10.1038/s41467-018-07461-x (PMC6269424; doi:10.1038/s41467-018-07461-x)
Supplement: Supplementary file 1 — Supplementary Information [file 41467_2018_7461_MOESM1_ESM.pdf]

Supplemental Information

Title: Adipocyte OGT governs diet-induced hyperphagia and obesity

Authors: Li et al.

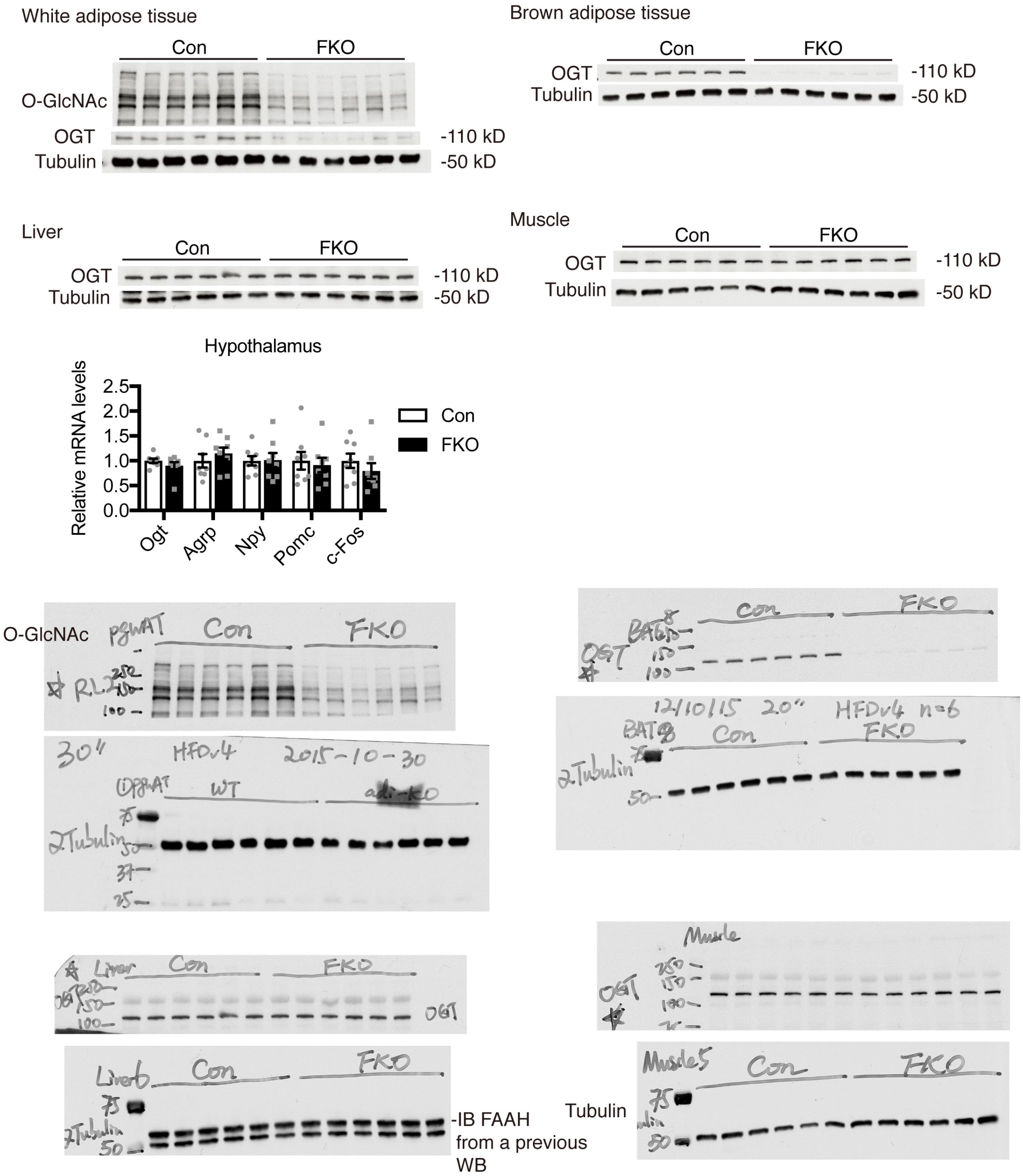

Supplementary Figure 1. Adiponectin-Cre ablates OGT in adipose tissues. Western blotting and real-time quantative PCR analysis of OGT in white adipose tissue, brown adipose tissue, liver, skeletal muscle, and hypothalamus (n = 6). Tissues were sampled from HFD-fed animals. Full blots are displayed on the lower panels. IB, immunoblot.

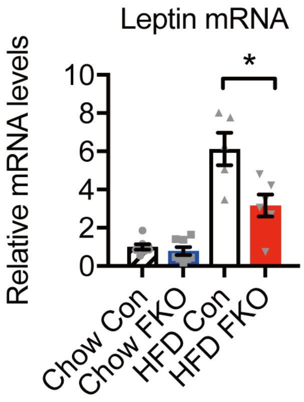

Supplementary Figure 2. Effects of OGT deletion on leptin expression. RT-qPCR analysis of leptin in white adipose tissue. Animal information is described in Fig. 1d. Data were presented as mean ± s.e.m. *$P < 0.05$, two-tailed unpaired Student's t-test.

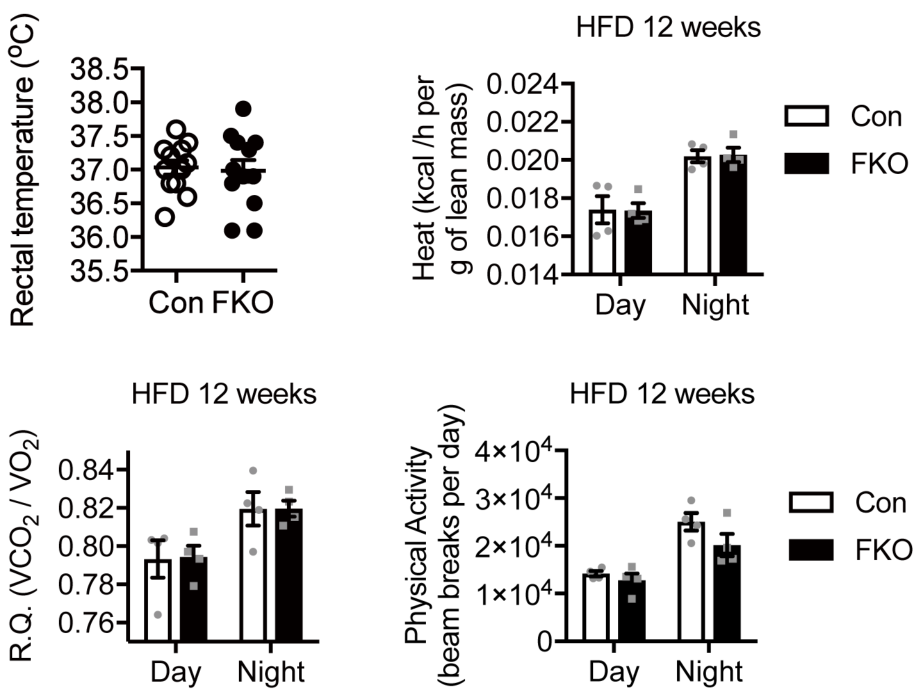

Supplementary Figure 3. Effects of OGT deletion on metabolic activities. Rectal temperature was recorded in Con (n = 12) and FKO (n = 12) mice that had been fed HFD for 18 weeks. Energy expenditure, respiratory quotient, and physical activity were measured in 12-week-HFD-fed Con (n = 4) and FKO (n = 4) animals. Data were presented as mean ± s.e.m. $^{*}P < 0.05$, $^{**}P < 0.01$, two-tailed unpaired Student's t-test.

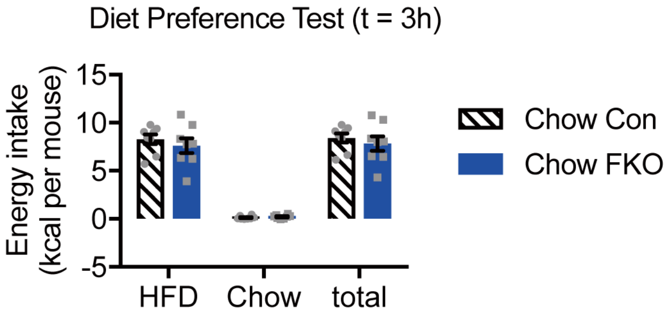

Supplementary Figure 4. Dietary preference is not altered in chow-fed FKO mice. 3-hour nocturnal energy intake in a two-choice test was measured in 22.5-week-old Con (n = 8) and FKO (n = 8) mice. Mice were acclimated by singly housing for 6 days and HFD feeding for 3 days. Data were presented as mean ± s.e.m. *$P < 0.05$, **$P < 0.01$, two-tailed unpaired Student's t-test.

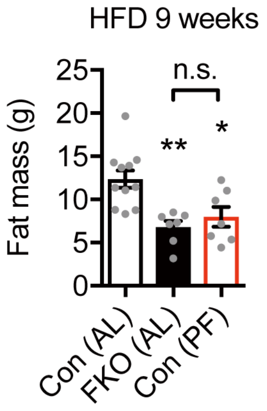

Supplementary Figure 5. Pair feeding normalized fat mass between FKO and Con mice. Con (AL, ad libitum, n = 11), Con (PF, pair-fed to littermate FKO, n = 7), FKO (AL, n = 7). Data were presented as mean ± s.e.m. *N.s.* not significant, *$P < 0.05$ (compared to Con (AL)), **$P < 0.01$ (compared to Con (AL)), *post-hoc* Tukey's test.

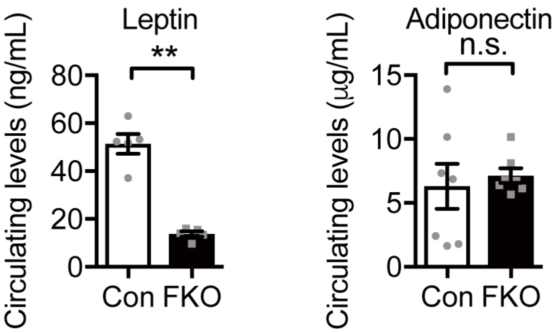

Supplementary Figure 6. Serum adipokines and lipid profiles in male Con (n = 7) and FKO (n = 7) mice fed HFD for 22 weeks. Data were presented as mean ± s.e.m. *N.s.* not significant, *P < 0.05, **P < 0.01, two-tailed unpaired Student's t-test.

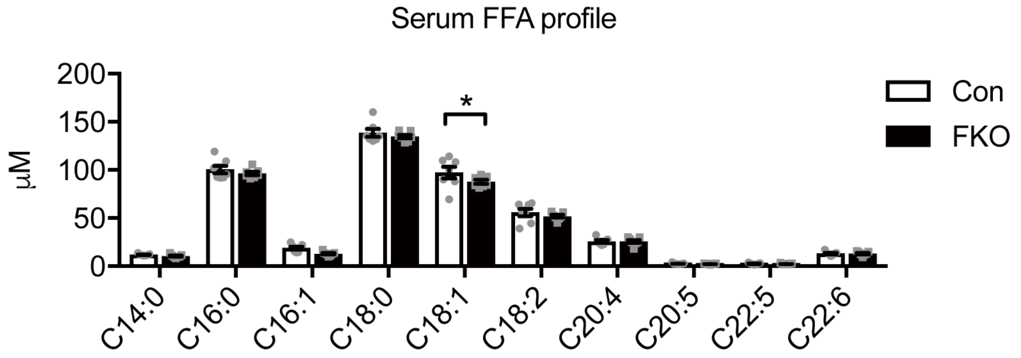

Supplementary Figure 7. Serum free fatty acid (FFA) profiles in male Con (n = 7) and FKO (n = 7) mice fed HFD for 22 weeks. Data were presented as mean ± s.e.m. *P < 0.05, *post-hoc* Sidak's multiple comparisons test.

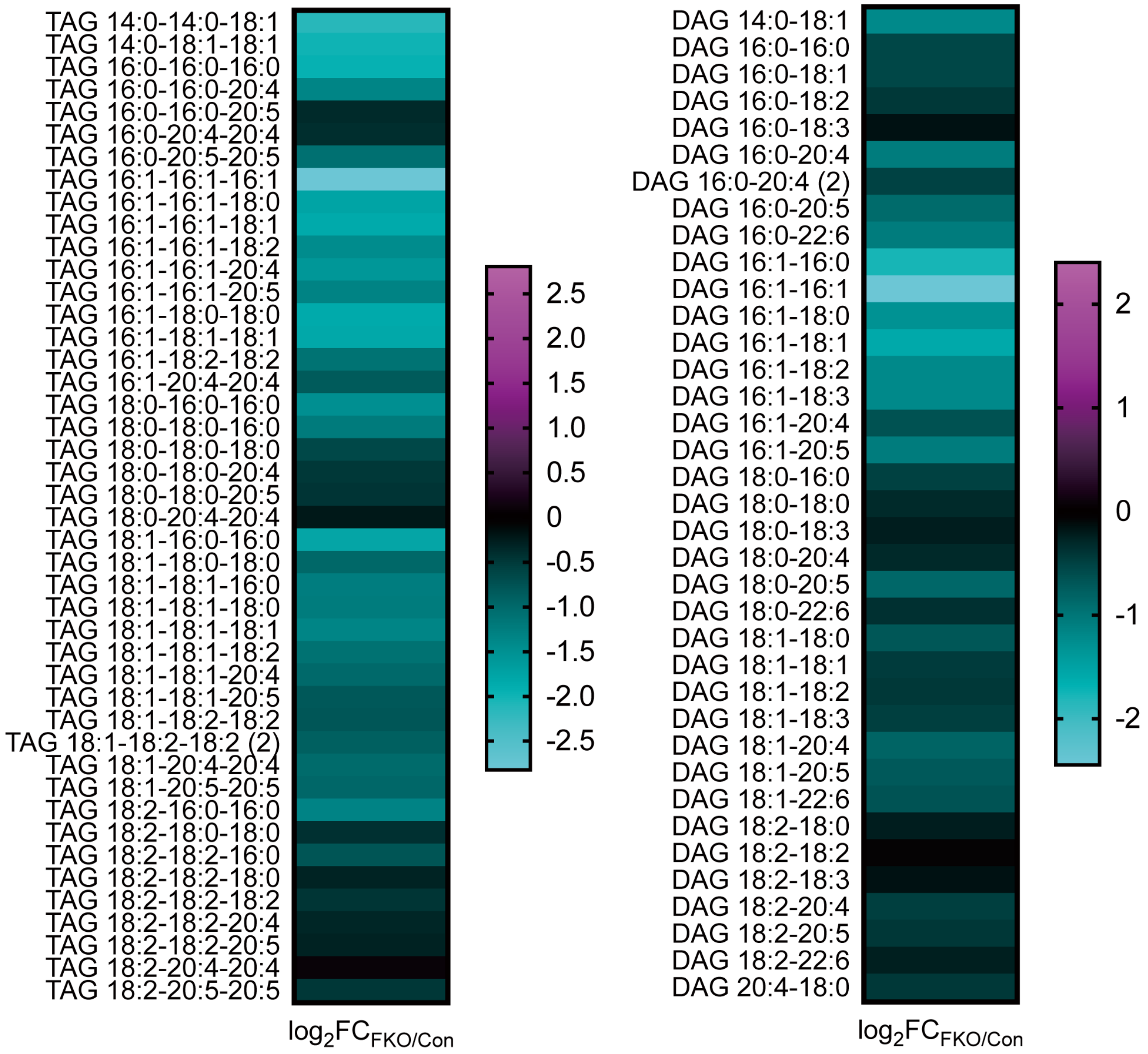

Supplementary Figure 8. Liver lipid profiles in male Con (n = 7) and FKO (n = 7) mice fed HFD for 22 weeks. Heatmaps show lipid profile of TAG (Left) and DAG (Right), respectively. Data were presented as the log2 ratio of the fold change (FC) of lipid levels in FKO vesus Con samples. A scale of $\log_2 FC_{FKO/Con}$ is presented on the right of the heatmap.

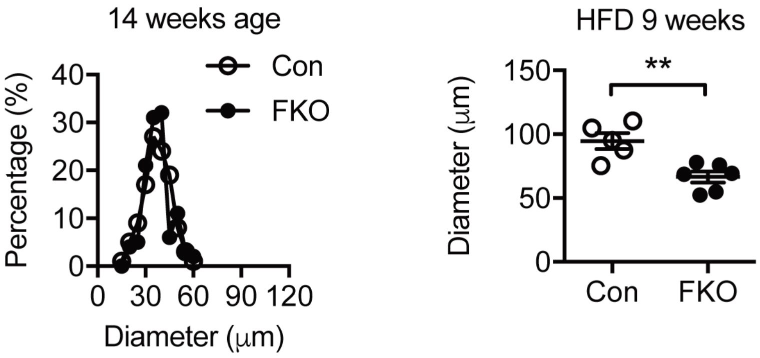

Supplementary Figure 9. Cell size analysis of histological sections of adipose tissue from 14-week-old chow-fed animals (Left) and 15-week-old 9-week-HFD-fed animals (Right). Data were presented as mean ± s.e.m. *P < 0.05, **P < 0.01, two-tailed unpaired Student's t-test.

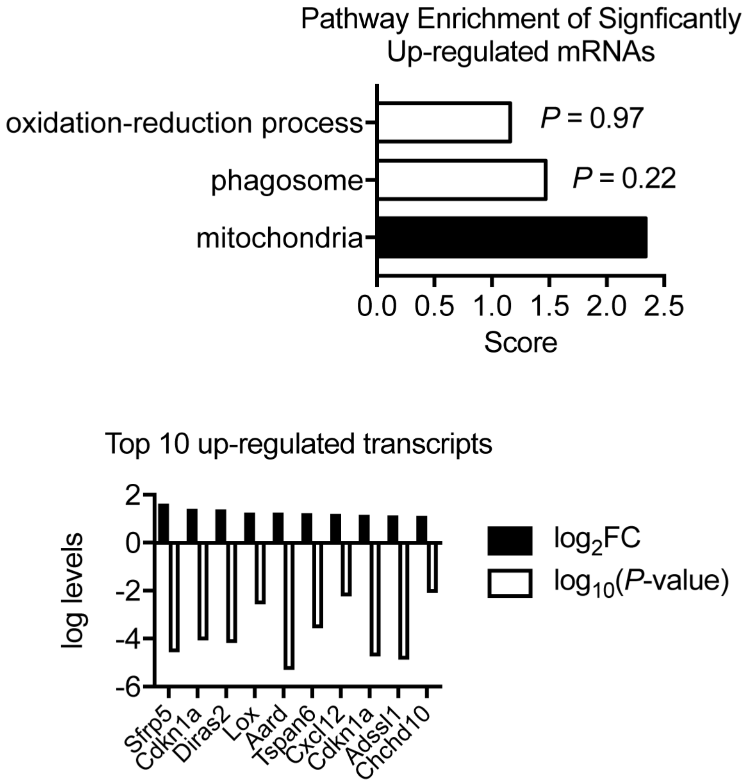

Supplementary Figure 10. Transcriptiomic analysis of adipose tissue. Upper graph shows the result of pathway enrichment analysis of perigonadal white adipose tissue (pgWAT) from male Con (n = 5) and FKO (n = 3) mice fed HFD for 3 days. Genes up-regulated by OGT depletion were analyzed in the DAVID 7.8 platform. Lower graph shows the list of top 10 up-regulated genes in adipose tissue. Data were presented as the log2 ratio of the fold change (FC) of signal levels in FKO and Con samples and the log10 ratio of the adjusted *P* value.

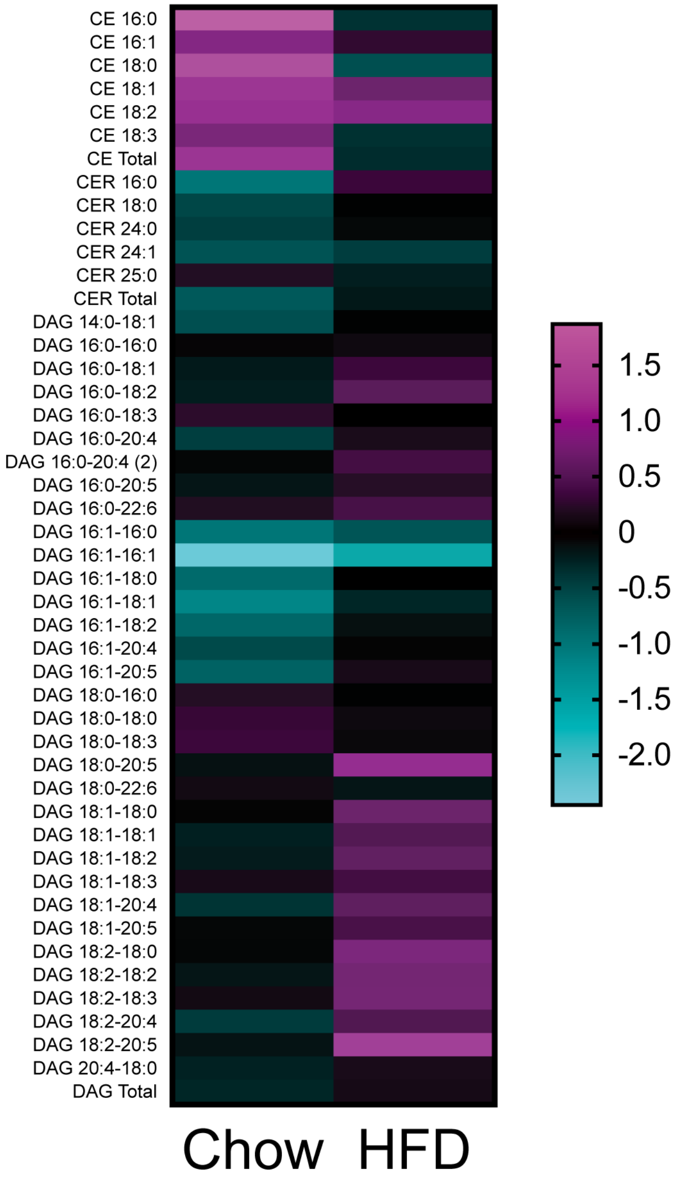

Supplementary Figure 11. Heatmap of lipid species in adipose tissue lipidome. CE, cholesterol ester, CER, ceramide, DAG, diacylglycerol. Data were presented as log2 ratios of FKO/Con (Chow for 14-15 weeks, Con n = 11, FKO n = 6; age-matched HFD for 9 weeks, Con n = 7, FKO n = 8).

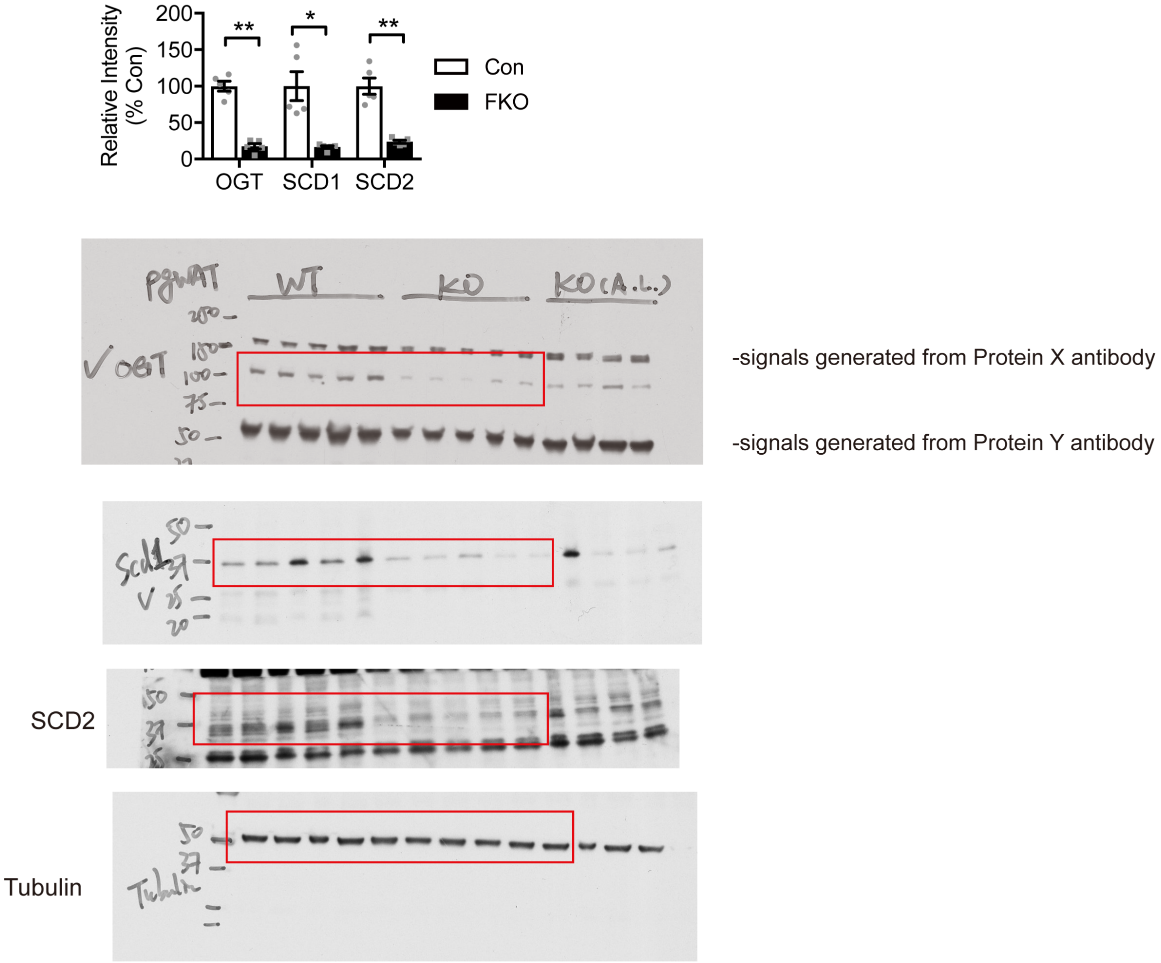

Supplementary Figure 12. Density graphs quantifying protein levels of SCD and OGT in adipose tissue. Data were presented as mean ± s.e.m. *P < 0.05, **P < 0.01, two-tailed Student's t-test. Full blots associated with Fig. 4b are displayed. Red frames indicate cropped region displayed in the main Fig. 4b.

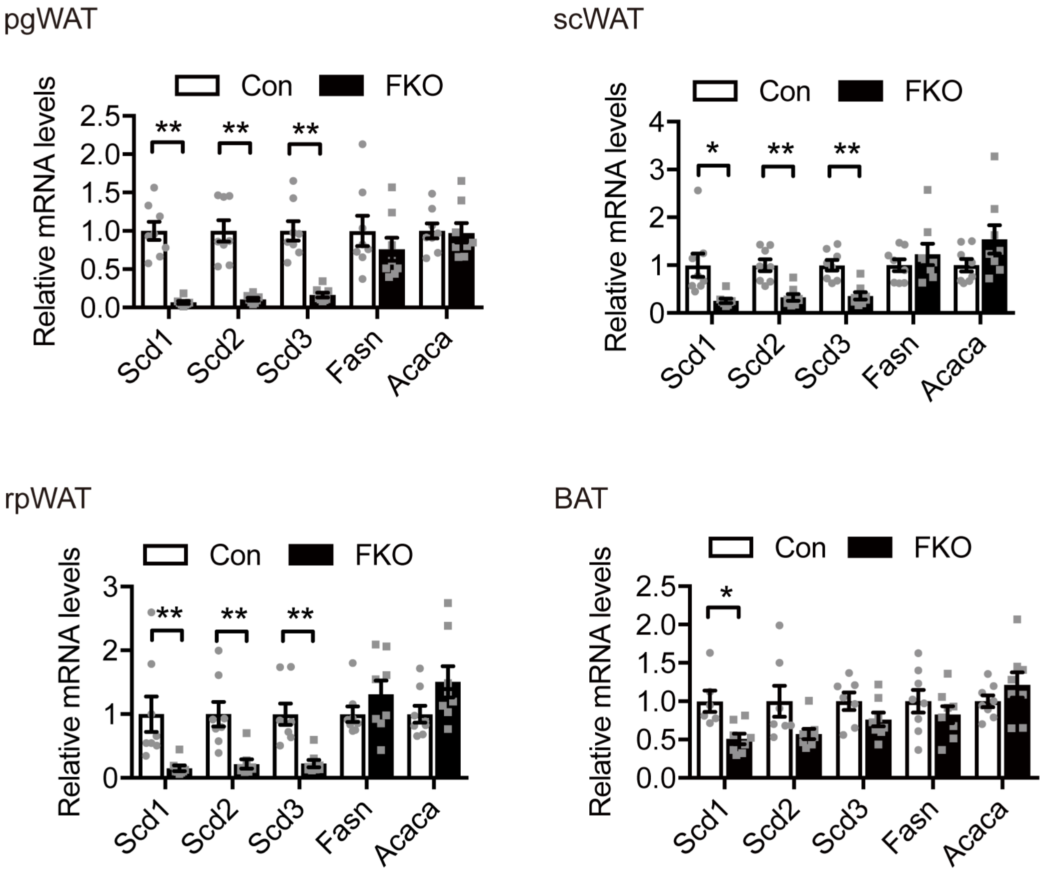

Supplementary Figure 13. Gene expression analysis of *de novo* lipid desaturation and synthesis in adipose depots. White adipose tissues from perigonadal (pg), subcutaneous (sc), and retroperitoneal (rp) regions, and interscapular brown adipose tissue (BAT) were collected from 15-week chow-fed Con (n = 8) and FKO (n = 8) mice. Data were presented as mean ± s.e.m. *$P < 0.05$, **$P < 0.01$, two-tailed unpaired Student's t-test.

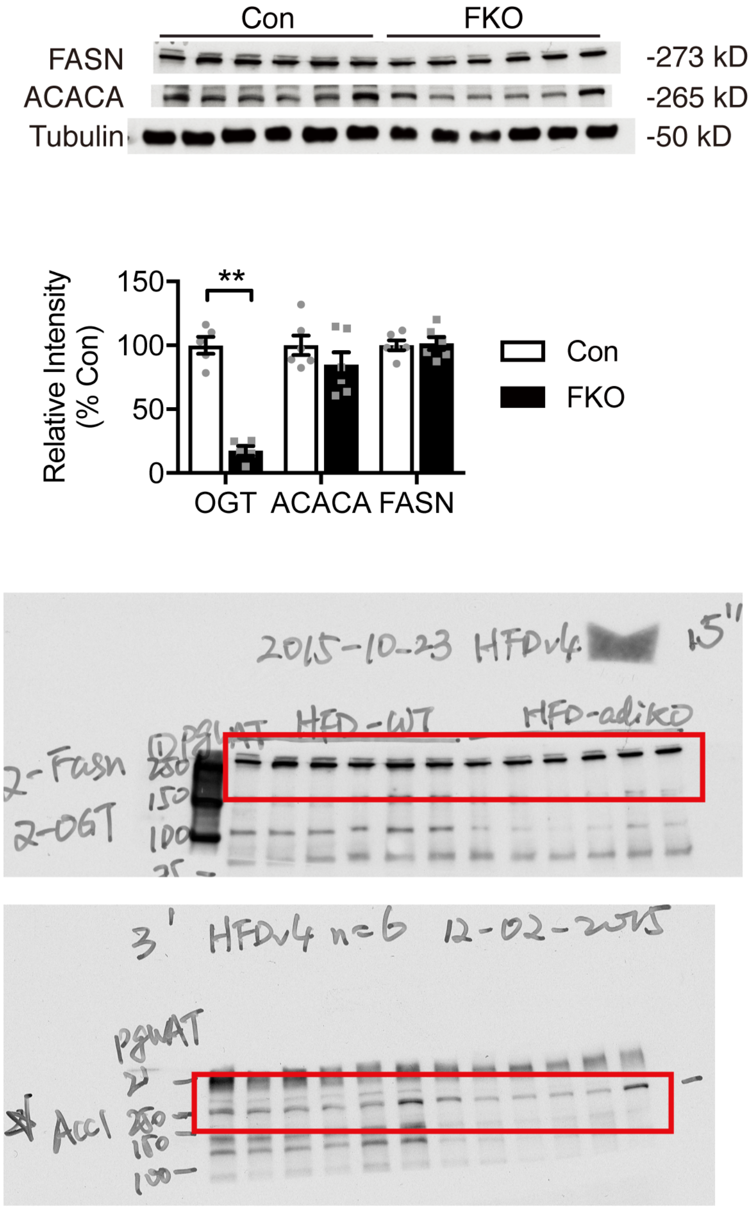

Supplementary Figure 14. Protein levels of Fasn and Acc1 are not altered by OGT deletion. Density graph displays the result of Western blot analysis of the pgWAT extracts from Con (n = 6) and FKO (n = 6) mice on HFD for 9 weeks. Data were presented as mean ± s.e.m. *$P < 0.05$, **$P < 0.01$, two-tailed unpaired Student's t-test. Full blots are displayed. Red frames indicate cropped region displayed in the top panel.

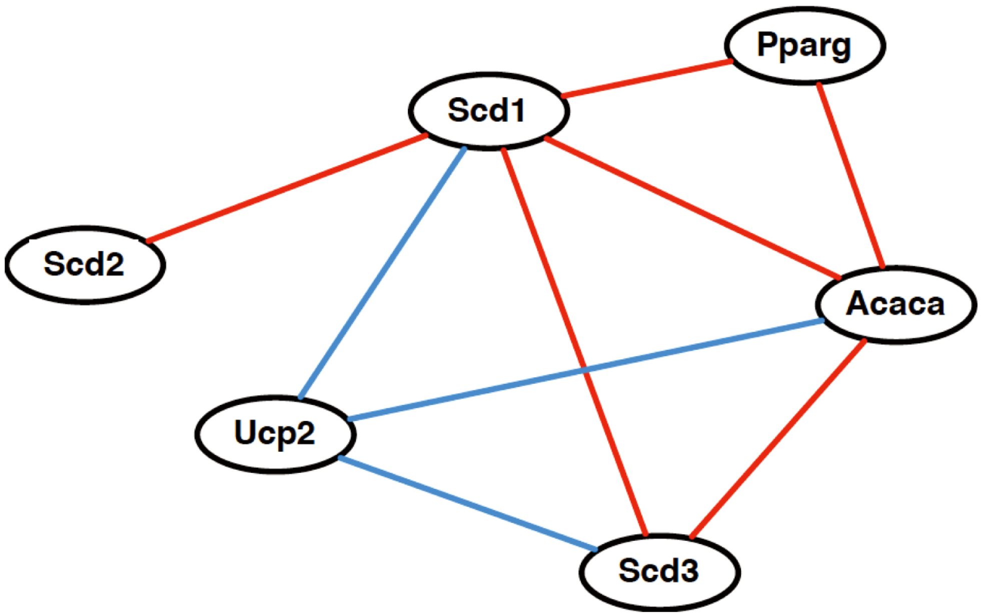

Data sources: the Hybrid Mouse Diversity Panel (HMDP)
http://www.ncbi.nlm.nih.gov/geo/query/acc.cgi?acc=GSE64768
Red line indicates positive correlation. Blue line indicates negative correlation

Supplementary Figure 15. Pparg and Scd genes are co-regulated at the transcript level. Gaussian graphical modeling was performed to estimate conditional interdependence among genes in *de novo* lipogenesis, Pparg, and Ucp2.

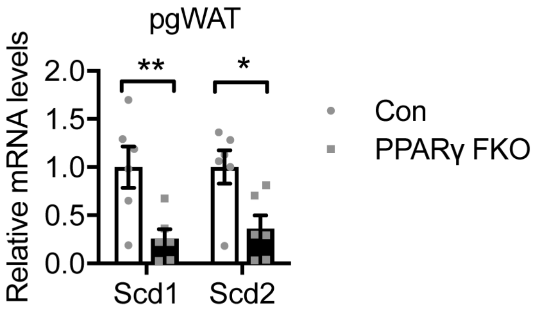

Supplementary Figure 16. Transcript levels of Scd1 and Scd2 in pgWAT from aP2 promoter-driven Cre PPARγ$^{flox/flox}$ (PPARγ FKO, n = 6) mice and littermate PPARγ$^{flox/flox}$ mice (Con, n = 6). Data were presented as mean ± s.e.m. *$P < 0.05$, **$P < 0.01$, two-way unpaired Student's t-test.

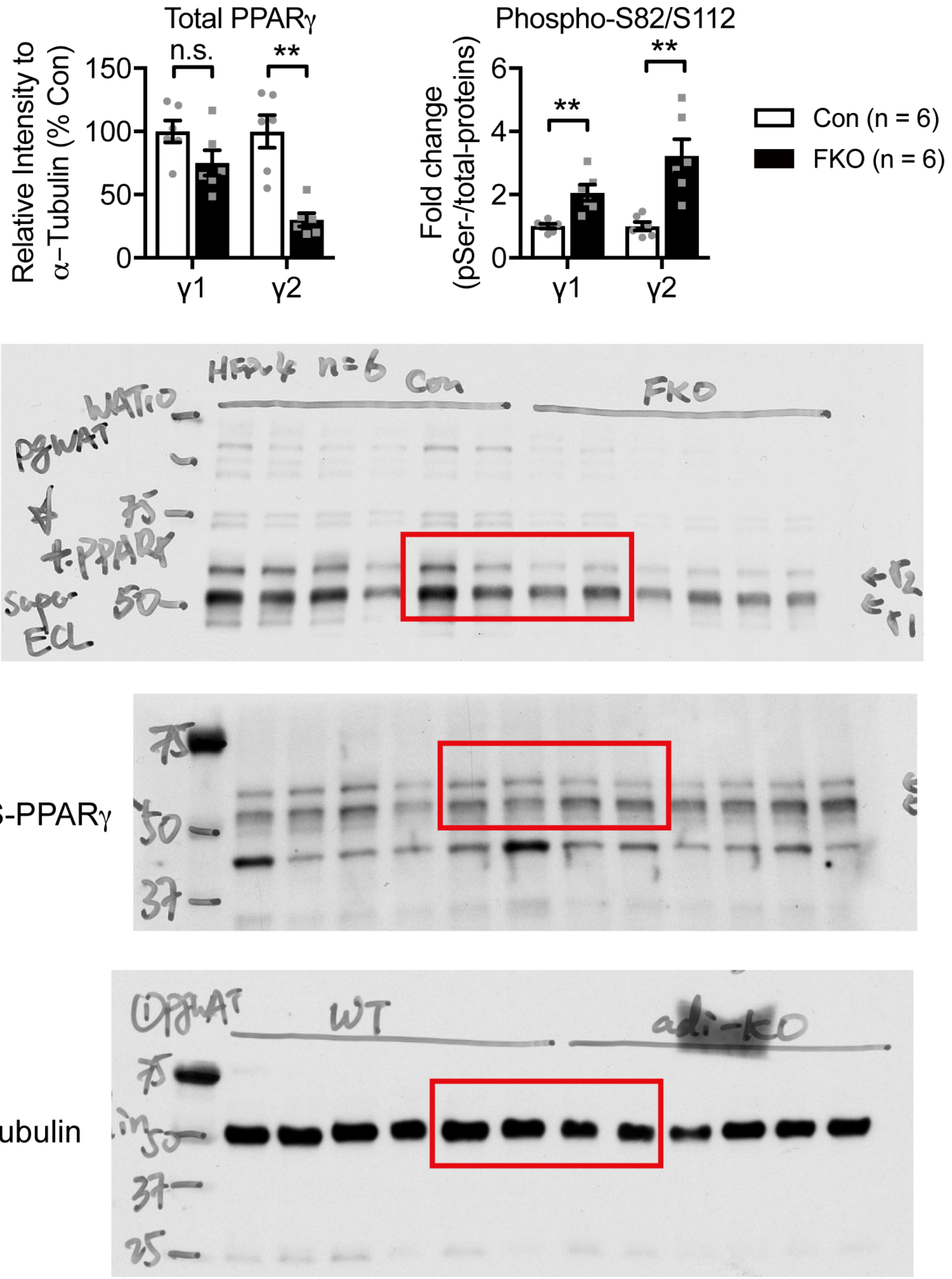

Supplementary Figure 17. Density graphs quantifying total or phosphorylated PPARγ proteins in adipose tissue from FKO and Con mice on HFD for 9 weeks (N = 6). Data were presented as mean ± s.e.m. **P* < 0.05, ***P* < 0.01, two-way unpaired Student's t-test. Full blots associated with Fig. 4c are displayed. Red frames indicate cropped region displayed in the main Fig. 4c.

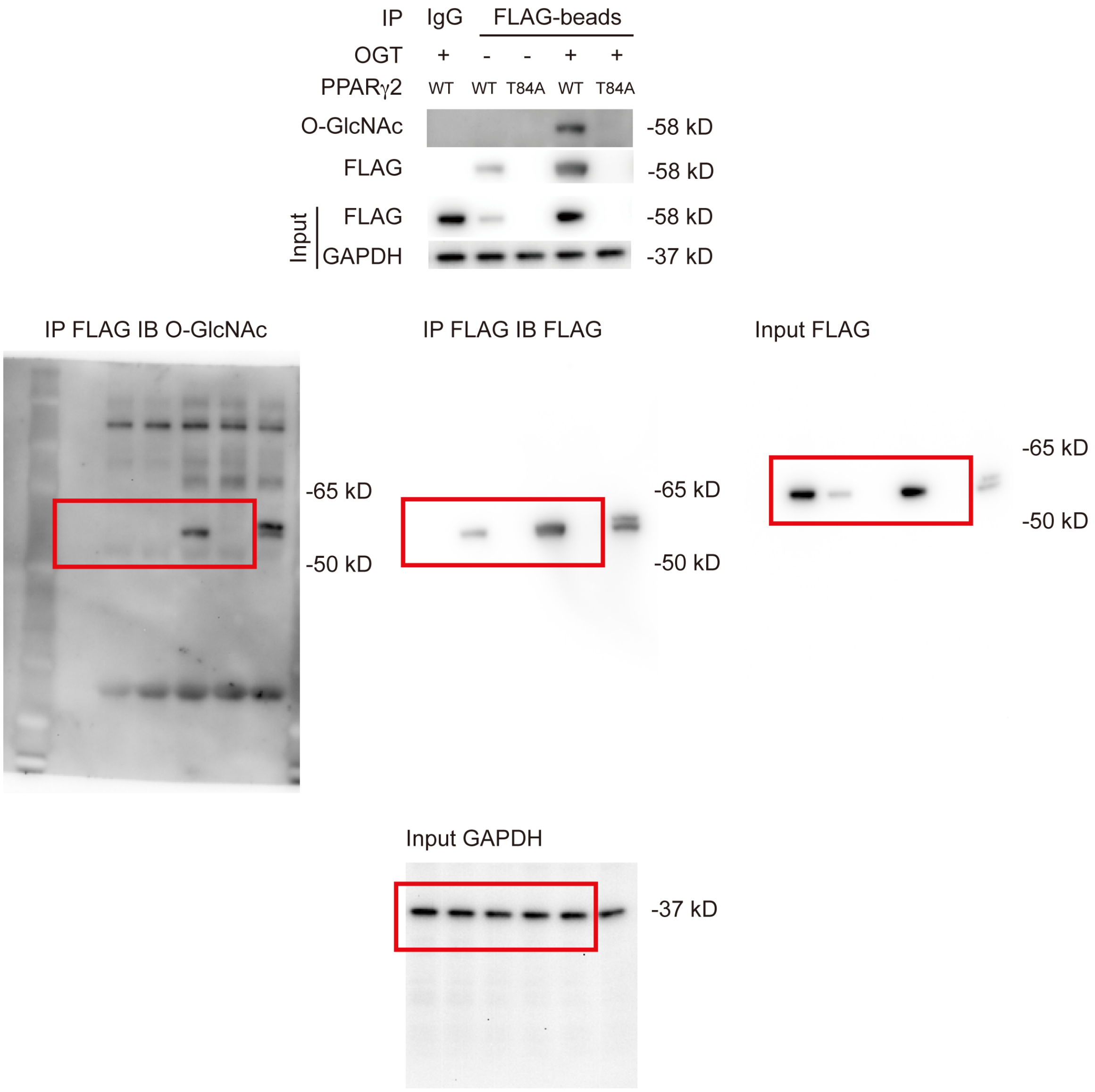

Supplementary Figure 18. Immunoprecipitation analysis of PPARγ2 O-GlcNAcylation in 293A cells. Experiments have been repeated for three times. Full blots associated are displayed. Red frames indicate cropped region displayed in the upper panel.

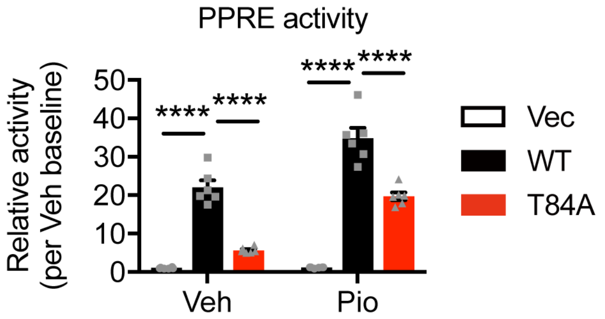

Supplementary Figure 19. Luciferase reporter assays of PPARγ2 mutants. Expression constructs for PPARγ2 and RXRα were co-expressed in 293A cells with a PPAR response elements (PPRE)-driven luciferase reporter (n = 6). Cells were treated with pioglitazone (Pio, 10 μM) overnight before the assays. Luminescence was normalized to Renilla reporter activity. Experiments have been repeated for three times. Data were presented as mean ± s.e.m. *$P < 0.05$, **$P < 0.01$, ****$P < 0.0001$, *post-hoc* Sidak's tests.

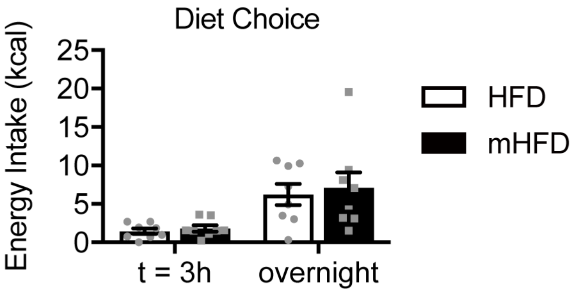

Supplementary Figure 20. Mice have no dietary preference between mono-unsaturated fat-fortified high fat diet (mHFD) and HFD. Energy intake (3h and 16h) in a two-choice diet preference test (n = 8). Data were presented as mean ± s.e.m. $*P < 0.05$, $**P < 0.01$, two-tailed unpaired Student's t-test.

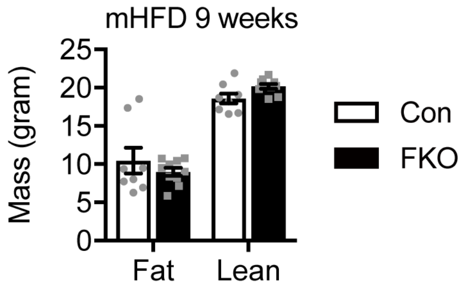

Supplementary Figure 21. Fat mass and lean mass in male Con (n = 8) and FKO (n = 10) mice fed mHFD for 9 weeks. Data were presented as mean ± s.e.m. *$P < 0.05$, **$P < 0.01$, two-tailed unpaired Student's t-test.

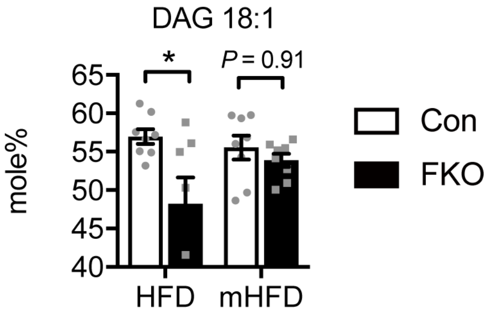

Supplementary Figure 22. Levels of oleoyl DAG in adipose lipidome, in mice fed HFD or mHFD for 15 weeks. Data were presented as mean ± s.e.m. **P* < 0.05, two-tailed unpaired Student's t-test.

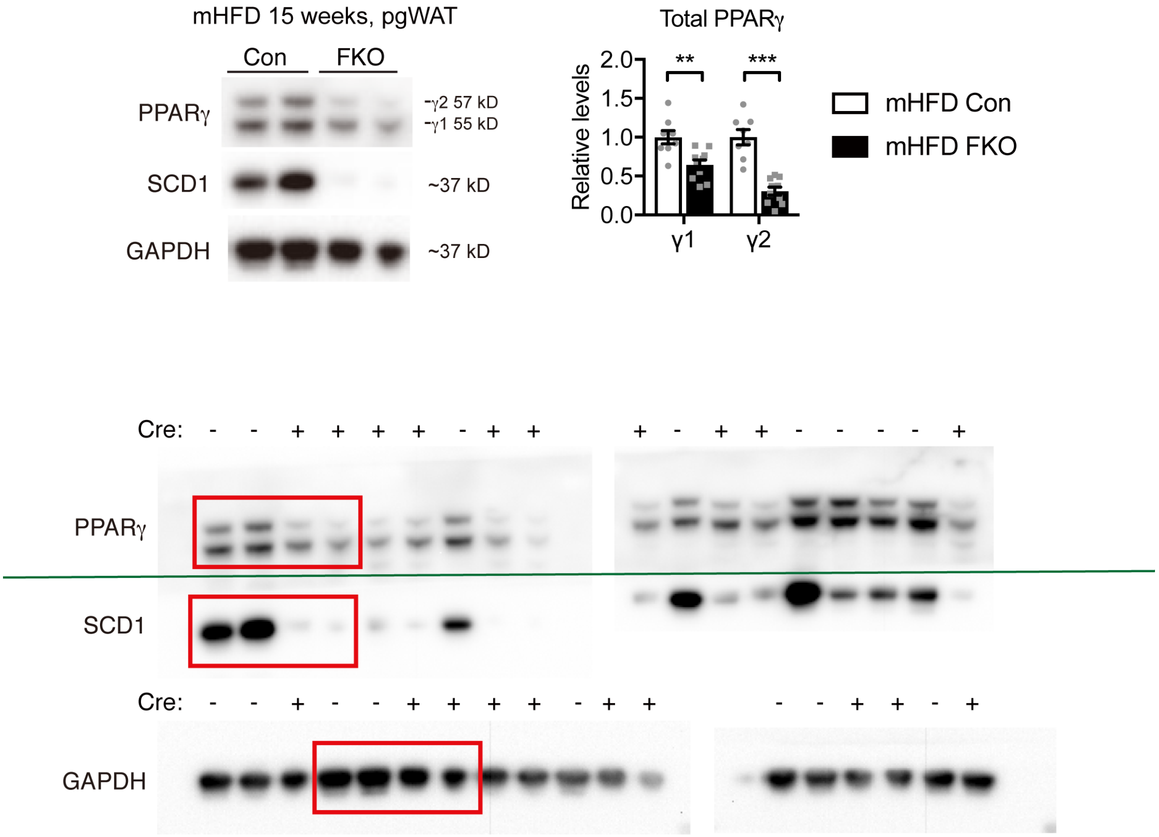

Supplementary Figure 23. Expression of PPARγ isoforms and SCD1 in mice on mHFD for 15 weeks (n = 8, Con; n = 10, FKO). Two representative mouse samples from each group were presented in Western blots. Data in the density graph were presented as mean ± s.e.m (Density signals of PPARγ normalized by those of GAPDH). *P < 0.05, **P < 0.01, ***P < 0.001, two-way ANOVA and *post-hoc* Sidak's multiple comparisons test. Full blots associated are displayed. Red frames indicate cropped region displayed in the upper panel.

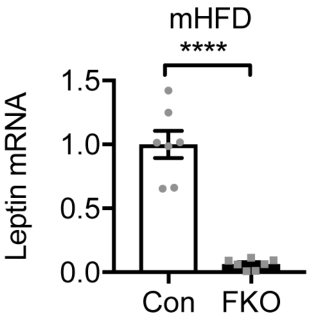

Supplementary Figure 24. Expression of leptin in mice on mHFD for 15 weeks (n = 7 per group). Data were presented as mean ± s.e.m. **P* < 0.05, ***P* < 0.01, *****P* < 0.0001, unpaired two-tailed Student's t-test.

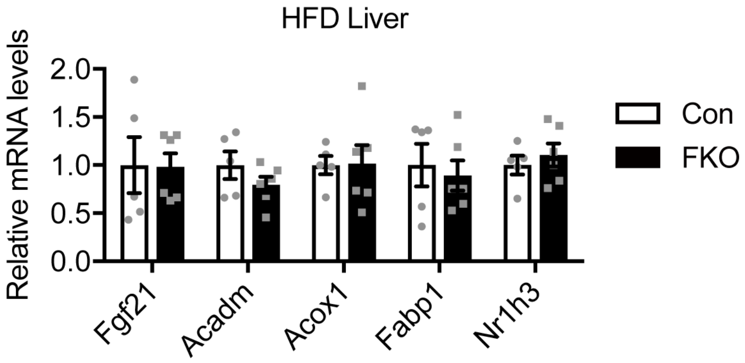

Supplementary Figure 25. Expression of PPARα target genes in liver from mice on HFD for 9 weeks. Data were presented as mean ± s.e.m. None of these genes were differentially regulated by FKO as assessed by unpaired two-tailed Student's t-test.

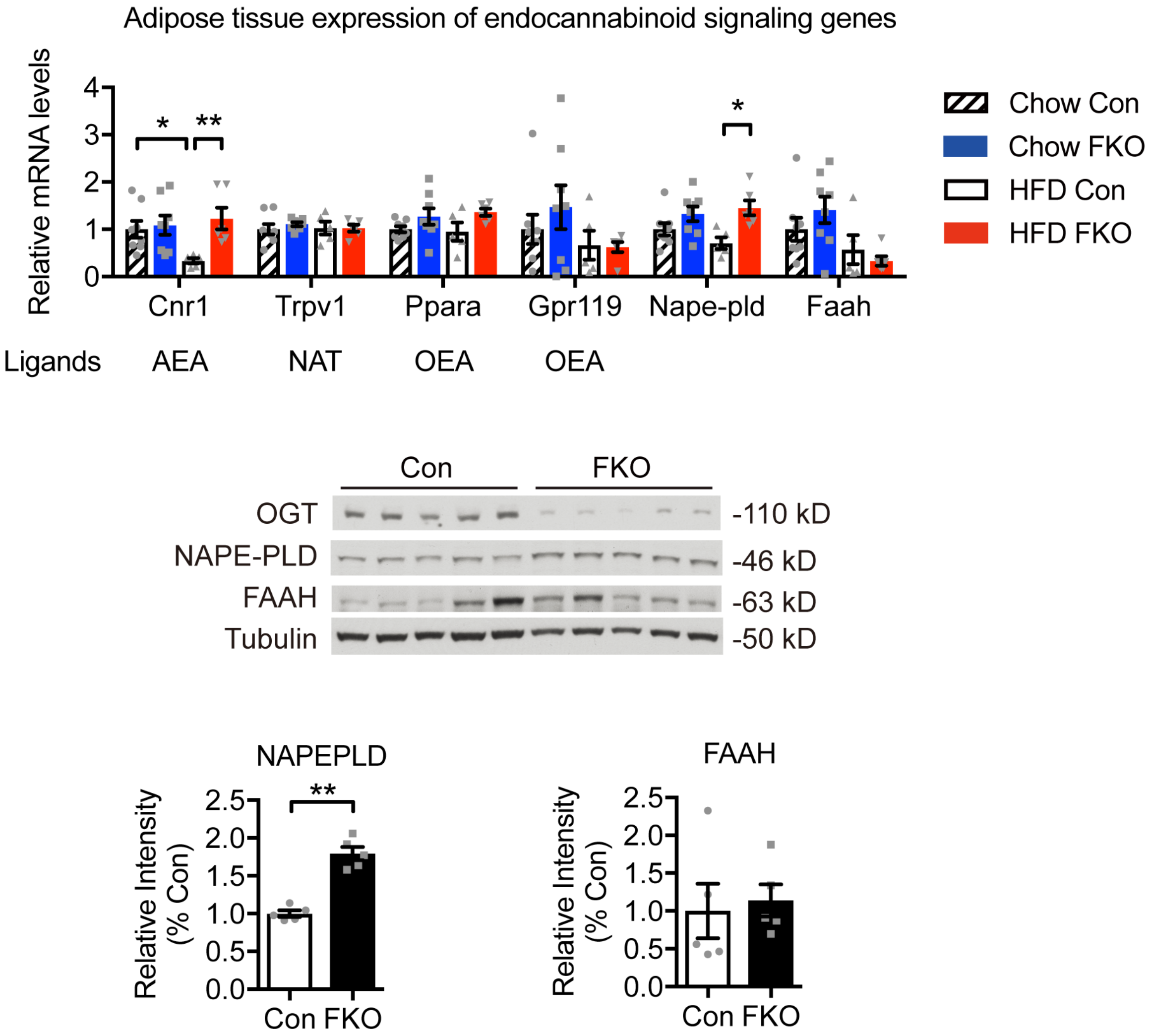

Supplementary Figure 26. Gene expression analysis of endocannabinoid signaling and metabolism in adipose tissue. Upper graphs show the mRNA levels in adipose tissue of chow-fed mice (n = 8 per group) and age-matched 9-week-HFD-fed mice (n = 5 (Con) or 6 (FKO)). Lower graphs show protein levels of NAPE-PLD and FAAH in adipose tissue from 9-week-HFD-fed mice (n = 5 per group). Data were presented as mean ± s.e.m. *$P < 0.05$, **$P < 0.01$, unpaired two-tailed Student's t-test. NAT, N-acyl taurine. Full blots associated are displayed in Supplementary Figure 27.

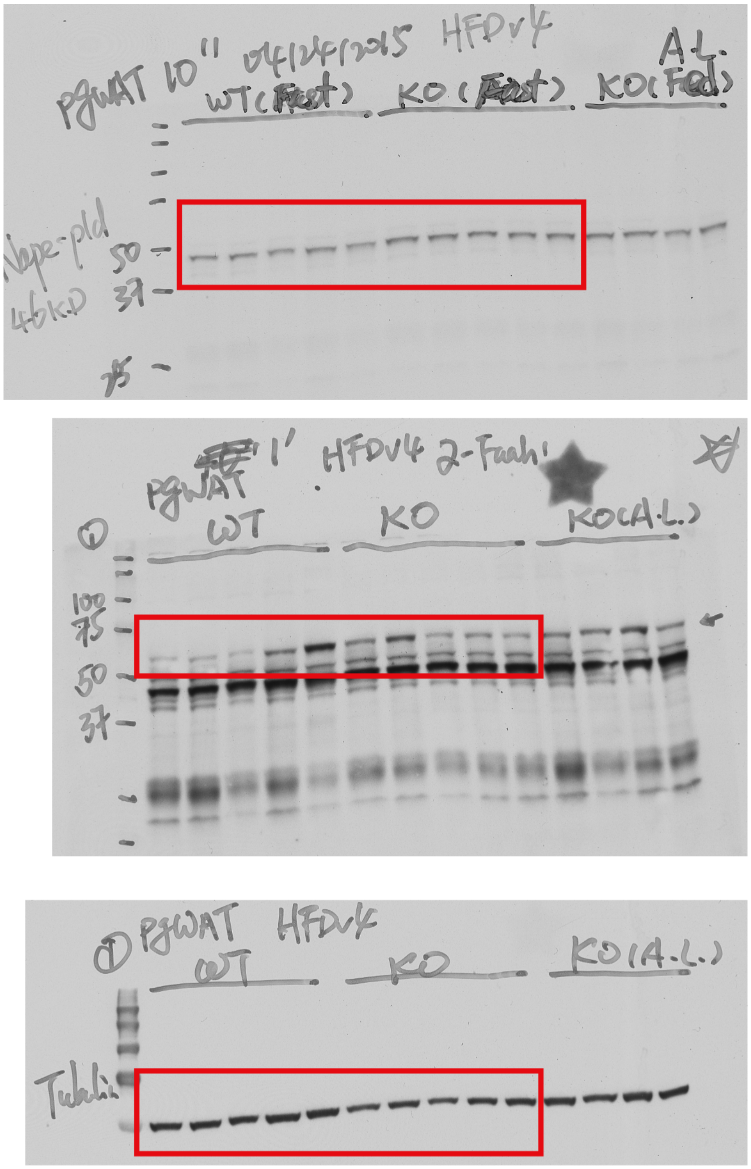

Supplementary Figure 27. Full blots associated with Supplementary Figure 26 are displayed. Red frames indicate cropped region displayed in the Supplementary Figure 26.

Supplementary Table 1. List of DNA Oligonucleotides

Oligo name	Forward	Reverse
u36B4	CCGATCTGCAGACACACACT	ACCCTGAAGTGCTCGACATC
Ogt	AAGAGGCACGCATTTTTGAC	ATGGGGTTGCAGTTCGATAG
Agrp	TGCTACTGCCGCTTCTTCAA	CTTTGCCCAAACAACATCCA
Npy	TAACAAGCGAATGGGGCTGT	ATCTGGCCATGTCCTCTGCT
Pomc	AGGCCTGACACGTGGAAGAT	AGGCACCAGCTCCACACAT
c-Fos	CCTTCGGATTCTCCGTTTCTCT	TGGTGAAGACCGTGTCAGGA
Scd1	CCGGAGACCCCTTAGATCGA	TAGCCTGTAAAAGATTTCTGCAAACC
Scd2	TGCCTTGTATGTTCTGTGGC	TCCTGCAAGCTCTACACCTG
Scd3	GCCTTGTACGTTCTGTGGCT	CCCTCCTGCAAGCTCTACAC
Acaca	GAAGCCACAGTGAAATCTCG	GATGGTTTGGCCTTTCACAT
Acly	AATGGCCGTCATGTGAGTTT	GTGGCCCCAACTATCAAGAG
Acss2	GCTGAACTGACACACCTGGA	AACTTGGCGACAAAGTTGCT
Fasn	GTTGGCCCAGAACTCCTGTA	GTCGTCTGCCTCCAGAGC
Elovl6	AACTTGGCTCGCTTGTTCAT	CCAATGGATGCAGGAAAACT
Srebf1	CTGTCTCACCCCCAGCATAG	GATGTGCGAACTGGACACAG
Mlxipl	CACTCAGGGAATACACGCCTAC	ATCTTGGTCTTAGGGTCTTCAGG
Nr1h3	TGGAGAACTCAAAGATGGGG	TGAGAGCATCACCTTCCTCA
Pparg	CAAGAATACCAAAGTGCGATCAA	GAGCTGGGTCTTTTCAGAATAATAAG
Lep	GACACCAAAACCCTCAT	CAGTGTCTGGTCCATCT
Fgf21	CTCCAGCAGCAGTTCTCTGA	CCTGGGTGTCAAAGCCTCTA
Acadm	ATGACGGAGCAGCCAATGA	ATGGCCGCCACATCAGA
Acox1	GGATGGTAGTCCGGAGAACA	AGTCTGGATCGTTCAGAATCAAG
Fabp1	CCAGGAGAACTTTGAGCCATTC	TGTCCTTCCCTTTCTGGATGA
Cnr1	TGAGAAAGAGGTGCCAGGAG	CTTGTAGCAGAGAGCCAGCC
Trpv1	CATCATCAACGAGGACCCAG	AACCAGGGCAAAGTTCTTCC
Ppara	AGTTCGGGAACAAGACGTTG	CAGTGGGGAGAGAGGACAGA
Gpr119	TTCCAGCACTTGGTAAAGGC	TGGGAGCTTCACTCTGTCCT
Nape-pld	TACTCATCCATGTCCCTCGG	ATATCTGCGTGGAACAGCCT
Faah	CTGTACCAGTTGGAGCAGGG	GAGACCATGGACAAGGCG
Pparg2-T84A	ccattgggtcagctcttgcgaatggaatgtcttcata	tatgaagacattccattcGcaagagctgacccaatgg
